# Methotrexate alleviates chronic inflammation in a *Drosophila* model

**DOI:** 10.1242/jcs.263816

**Published:** 2025-11-03

**Authors:** Dushyant K. Gautam, Willem Buys, Zeeshan Ahmad, Ravi K. Gutti, Indira Paddibhatla

**Affiliations:** ^1^ Department of Biochemistry, School of Life Sciences, University of Hyderabad, Hyderabad, TS 500046, India; ^2^ Cardiology Department, School of Medicine, Johns Hopkins University, Baltimore, MD 21201, USA; ^3^ Institute for Cell Engineering, Johns Hopkins School of Medicine, Baltimore, MD 21201, USA

**Keywords:** Inflammation, Pseudotumor, Tumorigenesis, Metabolism, Hemocytes

## Abstract

Growth signals and immune responses in cancer typically originate in the same compartments. In early stages of tumor development, inflammatory cells trigger responses against growing cancers. At a molecular level, it is unclear how the innate immune system recognizes tumorigenesis. At later stages, cancer cells resist cell death and evade immune detection, thereby suppressing anti-tumor responses and promoting cancer hallmarks. Often, chronic inflammatory responses become tumor friendly and incline towards tumorigenesis disturbing metabolic signaling, thereby rewiring nutritional supply for cancer growth. The precise connecting link between cancer, nutrition and metabolism remains unclear. *Drosophila* provides an ideal platform to explore the links between hyperactive immune signaling, defective fat metabolism and pseudotumor formation. Therefore, we examined the effects of methotrexate on these pathophysiological processes in larvae with hyperactive Toll/NF-κB pathway. We determined that both chemical (methotrexate) and genetic [rescue of *Ubc9*^−/−^ mutants by introducing a wild-type copy of Cactus (negative regulator of the Toll pathway)] interventions alleviated abnormalities associated with Toll/NF-κB hyperactivity and its influence on insulin signaling. Our study underscores drug repurposing studies and provides insights into how immune–metabolic crosstalk rewires inflammation-driven tumorigenesis.

## Teaser

**Summary:** Methotrexate reduces chronic inflammatory phenotypes due to hyperactive Toll, in evidence of its immunomodulatory potential independent of its folate agonist activity.

## INTRODUCTION

A fundamental aspect of cancerous growth is the inflammation-driven tumor microenvironment, with inflammation identified as one of the hallmarks of cancer (Greten and Grivennikov, 2019; Hanahan and Weinberg, 2000). In this context, detecting the escape mechanisms employed by tumor cells and the adaptive responses of non-cancerous cells to therapeutic agents becomes imperative (Spranger, 2016; Wu et al., 2021). Ongoing research not only emphasizes evaluation of the therapeutic efficacy of anti-cancer agents but also explores strategies to increase the resilience of non-cancerous cells, especially immune cells, to these interventions to initiate and sustain an efficient cancer immune response (Medler et al., 2015; Mukherjee et al., 2022; Tang et al., 2021; Zhang et al., 2022). We showed recently that methotrexate (MTX), a widely utilized chemotherapeutic drug, exerts anti-inflammatory effects against the hyperactive JAK/STAT pathway of *hop^Tum-l^ Drosophila* mutants (Yadav et al., 2021). Investigating the molecular mechanisms underlying this non-canonical drug effect in growing cancerous cells could help uncover potential treatment targets (Bedoui et al., 2019; Kozminski et al., 2020). In addition, research associated with the immunomodulatory properties of MTX could generate hypotheses to repurpose approved drugs against non-canonical targets and offer valuable insights into the molecular interplay between cancer treatment and immune regulation (Al Khzem et al., 2024).

**Fig. 1.**
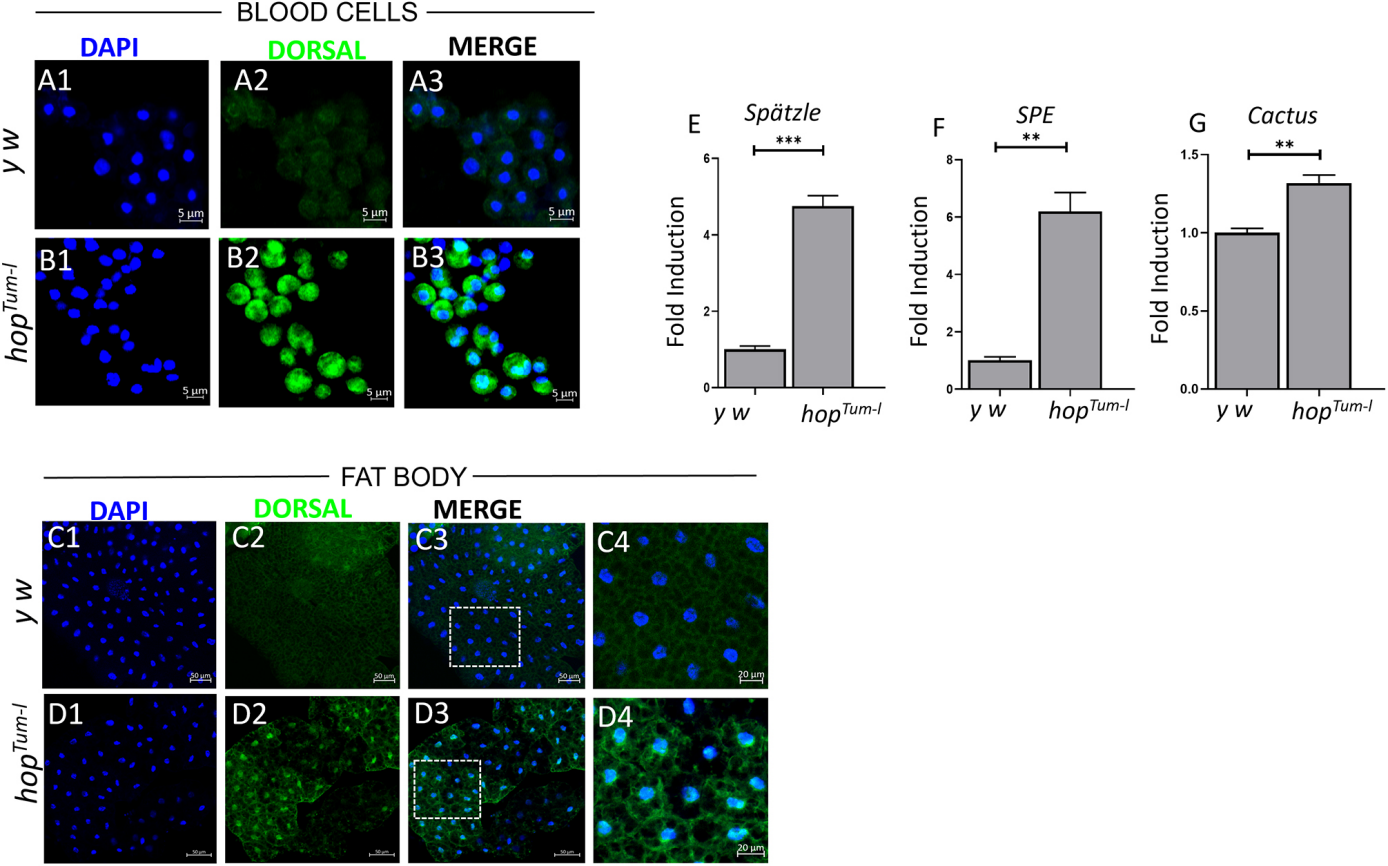
**Circulating blood cells in *hop^Tum-l^* mutants exhibit active Toll signaling.** (A1–B3,C1–D4) Dorsal nuclear localization in the immune tissues (circulating hemocytes, fat body) signifies activation of the Toll pathway. Third-instar *hop^Tum-l^* mutant larvae (*JAK/STAT* gain-of-function mutant larvae) display prominent dorsal (green) nuclear localization (B1–B3) within circulating hemocytes compared to control *y w* larvae (A1–A3). Similar dorsal nuclear localization was also reported in the fat body tissues of third-instar *hop^Tum-l^* larvae (D1–D4) compared to those of *y w* larvae (C1–C4). Data were accumulated from three biological replicates; hemolymph was analyzed from ten to 12 animals in each replicate (*N*=3, *n*=10–12). Control and experimental images were taken at identical settings in confocal microscopy (LSM710). (E–G) Relative gene expression of *spätzle* (E; ligand), *SPE* (F; positive regulator) and *Cactus* (G; negative regulator) components of the Toll/NF-κB pathway was analyzed in *hop^Tum-l^* mutants and compared with that in the *y w* control whole larvae. Data from three biological repeats with more than 50 animals for each replicate were represented (*N*=3, *n*=50+). Student's *t*-test (unpaired, two-tailed); **P<0.01, ***P<0.001. Bar graphs were processed using GraphPad Prism version 8.0.2.

Understanding the mechanism of action behind the anti-cancer effects of MTX in *Drosophila* and comparing them to the effects observed in the current study can provide valuable insights into the benefits of drug repositioning and repurposing studies. The cytotoxicity observed in cells due to MTX by blocking folate-dependent DNA synthesis is already known (Kozminski et al., 2020; Rajagopalan et al., 2002; Worm et al., 2001). In addition to interrupting the folate pathway, MTX also has a non-canonical impact on immune signaling pathways such as the NF-κB and JAK/STAT pathways (Bedoui et al., 2019; Thomas et al., 2015; Yadav et al., 2021). NF-κB is the focal point of many inflammatory pathways (Baldwin, 1996), including the Toll-like receptor pathway (Bick et al., 2022; Zhang and Ghosh, 2001), and frequently interacts with other inflammatory pathways, such as JAK/STAT (Guo et al., 2024; Haftcheshmeh et al., 2022). Owing to high conservation of the Toll/Toll-like receptor pathway across distant species, particularly between humans and *Drosophila melanogaster*, it has been an invaluable paradigm to interrogate cellular inflammation (Valanne et al., 2011). We hypothesized that the DNA damage-independent anti-inflammatory effects of MTX are suppressing immune responses (both cellular and systemic) triggered in larval backgrounds of *Drosophila* with hyperactive Toll pathway. We, therefore, investigated the impact of MTX on the constitutively activated Toll pathway in *Drosophila* larvae exhibiting defective hematopoiesis and dysregulated inflammatory responses. Earlier, we showed that MTX impedes chronic inflammatory phenotypes observed in *hop^Tum-l^* mutants (Yadav et al., 2021). In the current study, to determine whether the Toll pathway is upregulated in JAK/STAT mutants, we first analyzed *hop^Tum-l^* mutants. Immunostaining revealed nuclear translocation of Dorsal (*Drosophila* NF-κB homolog) in blood cells (Fig. 1B1–B3) and fat body cells (Fig. 1D1–D4) of *hop^Tum-l^* mutants, whereas wild-type *y w* larval cells showed predominantly cytoplasmic Dorsal localization (Fig. 1A–A3,C1–C4). Elevated transcript levels of *spätzle*, *Spätzle-Processing Enzyme* (*SPE*) and *Cactus* (a negative regulator of the Toll pathway) (Fig. 1E–G) further confirmed concomitant Toll pathway activation in hyperactive JAK/STAT mutants. These results indicated that our hypothesis could be investigated to elucidate the effects of MTX on Toll pathway-dependent inflammatory responses, uncontrolled hematopoietic expansion and morphological changes in the fat body.

## RESULTS

### MTX downregulates acute inflammation in parasite-infected hosts

**Fig. 2.**
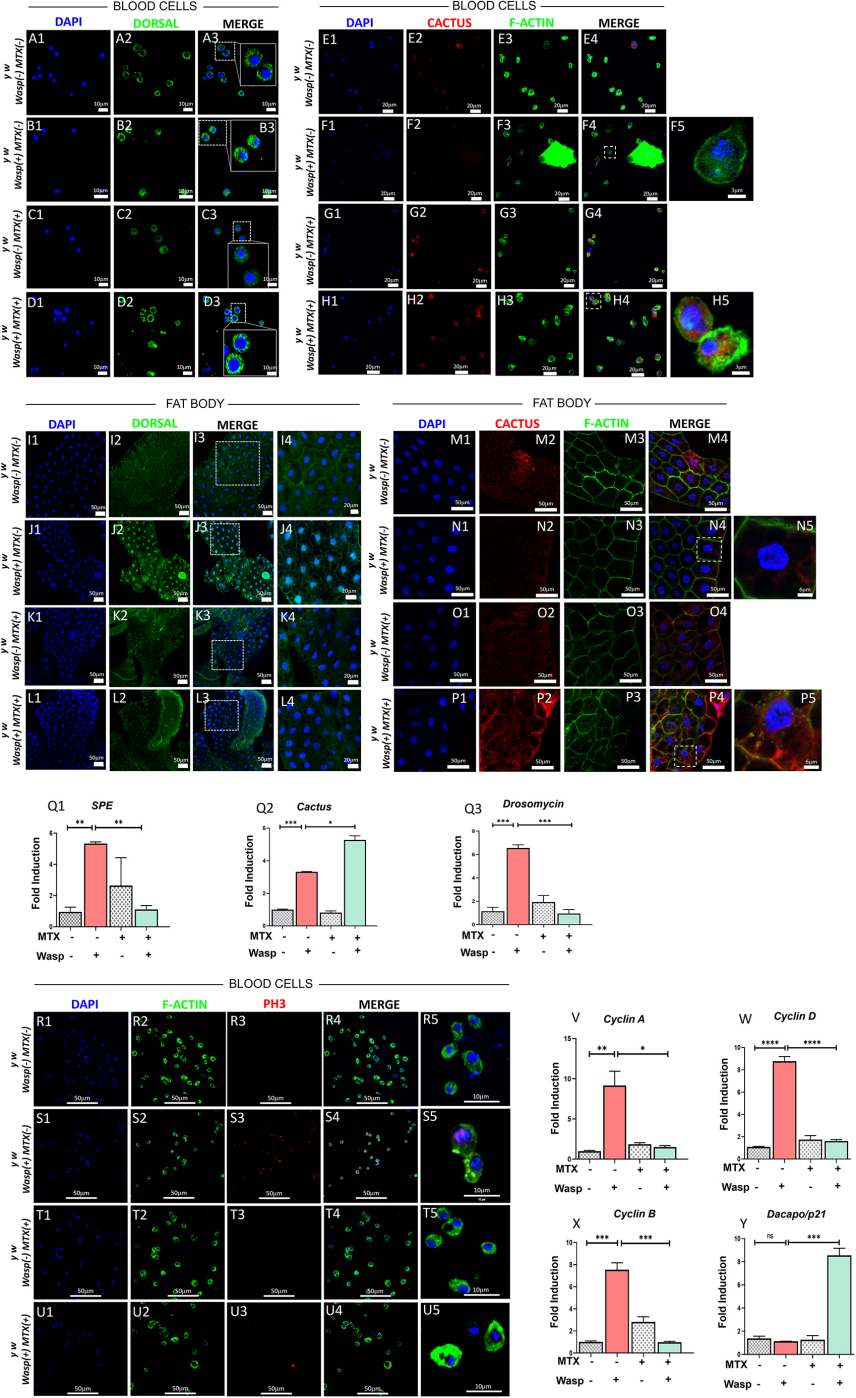
**Effect of methotrexate on Toll pathway induced by *Lb-17* parasitoid wasp infestation.** The larval host initiates an inflammatory response in the immune tissues (blood cells, fat body) by upregulating one of the immune signaling pathways (Toll pathway) against parasitoid infestation. Dorsal nuclear localization was investigated in circulating hemocytes and fat body cells of *y w* third-instar larvae with and without methotrexate (MTX) treatment post *Lb-17* parasitoid wasp infestation. Samples were stained with DAPI (nuclear stain, blue) and anti-Dorsal (Dorsal-specific antibody, green) to assess subcellular localization. (A1–D3) The first batch of third-instar larvae were dissected for circulating hemocytes in drug-free *y w* uninfected animals (A1–A3), stained for anti-Dorsal (A2), co-labelled with DAPI (A1). Drug-free *y w* animals infested by wasps (B1–B3) served as positive control. (C1–C3) Negative control devoid of wasp infestation but drug treated. (D1–D3) Larvae with wasp infestation post drug treatment. Data were obtained from three biological replicates (*N*=3, *n*=12), and all confocal microscopy images were acquired under identical settings using an LSM710 system. (I1–L4) Similarly, the second batch of larvae were dissected for assessment of Dorsal localization in fat body cells in drug-free *y w* uninfected animals (I1–I4), wasp-infested drug-free animals (J1–J4), uninfected but drug-treated animals (K1–K4) and wasp-infected drug-treated animals (L1–L4). Data were collected from three biological replicates, with hemolymph analyzed from 10–12 larvae per experimental group per replicate (*N*=3, *n*=10–12). Control and experimental images were acquired under identical settings using confocal microscopy (LSM710). Cactus, a negative regulator of the Toll pathway, sequesters Dorsal in the cytoplasm, preventing its nuclear translocation under normal conditions. To assess the impact of MTX treatment on Toll pathway modulation, Cactus protein localization was analyzed in circulating hemocytes and fat body cells. Samples were stained with DAPI (nuclear stain, blue), anti-Cactus (Cactus-specific antibody, red) and polymerized F-actin (cytoskeleton, green). (E1–H5) Cactus localization in hemocytes in untreated and uninfected (E1–E4), wasp-infected and untreated (F1–F5), uninfected and MTX-treated (G1–G4), and infected and MTX-treated (H1–H5) larvae. (M1–P5) Cactus localization in fat body cells in untreated and uninfected (M1–M4), wasp-infected and untreated (N1–N5), uninfected and MTX-treated (O1–O4), and infected and MTX-treated (P1–P5) larvae. Data were collected from three biological replicates (*N*=3, *n*=12), and all confocal microscopy images were acquired under identical settings using an LSM710 system. (Q1–Q3) RT-PCR analysis was used to quantify the expression of key Toll pathway components, including *SPE* (Q1), *Cactus* (Q2) and *Drosomycin* (Q3). Student's *t*-test (unpaired, two-tailed) showed *P<0.05, **P<0.01, ***P<0.001 when comparing MTX untreated with uninfected, infected and infected MTX-treated groups. (R1–U5) Cell proliferation and cell cycle regulation were analyzed in *y w* third-instar whole larvae under different infection and treatment conditions to assess the effect of MTX on wasp infection-induced Toll/NF-κB pathway activation. Actively proliferating cells were identified using anti-phospho-histone H3 (PH3; red), with DAPI (nuclear stain, blue) and polymerized F-actin (cytoskeleton, green) marking nuclear and cytoskeletal structures, respectively. Conditions analyzed include untreated and uninfected (R1–R5), wasp-infected and untreated (S1–S5), uninfected and MTX-treated (T1–T5), and infected and MTX-treated (U1–U5). Confocal images were acquired under identical settings using an LSM710 microscope (*N*=3, *n*=12). (V–Y) Gene expression studies of cell cycle regulators in whole larvae via RT-PCR analysis quantified the expression of *Cyclin A* (V), *Cyclin B* (W) and *Cyclin* D (X) (positive regulators of cell cycle progression), as well as *dacapo* (*p21*; a cell cycle inhibitor; Y) under different experimental conditions. Student's *t*-test (unpaired, two-tailed) showed *P<0.05, **P<0.01, ***P<0.001, ****P<0.0001 when comparing MTX untreated with uninfected, infected and infected MTX-treated groups. ns, not significant. Data were obtained from three biological replicates (*N*=3, *n*=50+), and graphs were generated using GraphPad Prism version 8.0.2. Data from three biological repeats with more than 50 animals for each replicate were represented for gene expression studies (*N*=3, *n*=50+).

In response to wasp infection in third-instar larvae, the Toll/NF-κB pathway, essential for blood cell proliferation and immune defense, is triggered (Paddibhatla et al., 2010; Qiu et al., 1998). Increased blood cell division is essential for encapsulation of the wasp egg, thereby controlling the infestation (Anderl et al., 2016). We first studied the impact of MTX on Toll pathway activity in wild-type *y w* larvae infected with *Leptopilina boulardi*-*17* (*Lb-17*) wasps (a model used to study acute inflammatory responses) (Paddibhatla et al., 2010). Four experimental groups were examined: uninfected larvae, infected larvae, and infected larvae treated with or without MTX. Encapsulation, a measure of immune response against wasp infestation, was reduced by 43.10% in MTX-treated larvae compared to 68.06% in untreated larvae (Fig. S1A). Subsequent qualitative analysis of Dorsal localization via immunostaining in blood cells and the fat body of these animals revealed no difference in Dorsal localization in uninfected larvae in the absence of MTX and post treatment with MTX (Fig. 2A1–A3,C1–C3,I1–I3,K1–K3) and increased nuclear Dorsal expression in infected larvae (Fig. 2B1–B3,J1–J3), which was reduced by MTX treatment (Fig. 2D1–D3,L1–L3). As the immunostaining results showed both cytoplasmic and nuclear expression of Dorsal, we determined the percentage of cells with nuclear versus cytoplasmic expression of Dorsal protein. Quantitative analysis indicated that 93.85% of fat body cells and 66.69% of hemocytes in infected larvae exhibited nuclear Dorsal, which decreased to 15.53% and 35.34%, respectively, upon MTX treatment (Fig. S1B1,B2). We next examined the effects of MTX on the transcript levels of components of the Toll pathway – *SPE*, *Drosomycin* and *Cactus*. We observed that, in infected larvae without MTX treatment, transcript levels of *SPE*, *Drosomycin* (the target of the pathway) and *Cactus* were significantly increased compared to those in uninfected controls, indicating possible upregulation of the Toll pathway post wasp infection (Fig. 2Q1–Q3). But, in infected larvae, whereas *SPE* and *Drosomycin* transcript levels showed downregulation (Fig. 2Q1,Q3) in response to MTX treatment, the levels of *Cactus* increased (Fig. 2Q2). We confirmed these results by immunostaining for Cactus, a Toll pathway inhibitor, the levels of which increased from 20.55% to 32.62% (Fig. S1C) in MTX-treated infected larvae, without notable effects on uninfected larvae (Fig. 2G1–G4,O1–O4).

Toll signaling modulates cell cycle progression by regulating the expression of cell cycle regulators (Hasan et al., 2005). Therefore, we also assessed the effects of MTX on cell cycle regulators by studying transcript levels of *Cyclin A*, *Cyclin B* and *Cyclin D*, along with those of the cell cycle inhibitor *dacapo* (*p21*) by quantitative reverse transcription PCR (qRT-PCR) in our acute inflammation model. In wasp-infected larvae, MTX treatment downregulated *Cyclin A*, *B* and *D* expression, while upregulating *dacapo* expression (Fig. 2V–Y). No significant changes in the expression levels of *Cyclin A* and *Cyclin D* were observed in uninfected untreated and treated larvae (Fig. 2V,W,Y), but the expression of *Cyclin B* was increased significantly post MTX treatment (Fig. 2X). We next assessed mitotic activity to determine whether cell division was compromised due to differential expression of cell cycle regulators. We observed that MTX treatment resulted in a reduced mitotic index, with only 61.50% of blood cells being phospho-histone H3 positive, compared to 87.30% in untreated larvae (Fig. S1D). Taken together, these results indicate that MTX effectively suppresses the Toll/NF-κB signaling pathway, attenuating the acute inflammatory responses induced by *Lb-17* wasp infestation, and this inhibits inflammation-driven cell cycle progression.

### MTX mitigates the hematopoietic defects manifested by overexpression of *SPE* in larval immune tissues

**Fig. 3.**
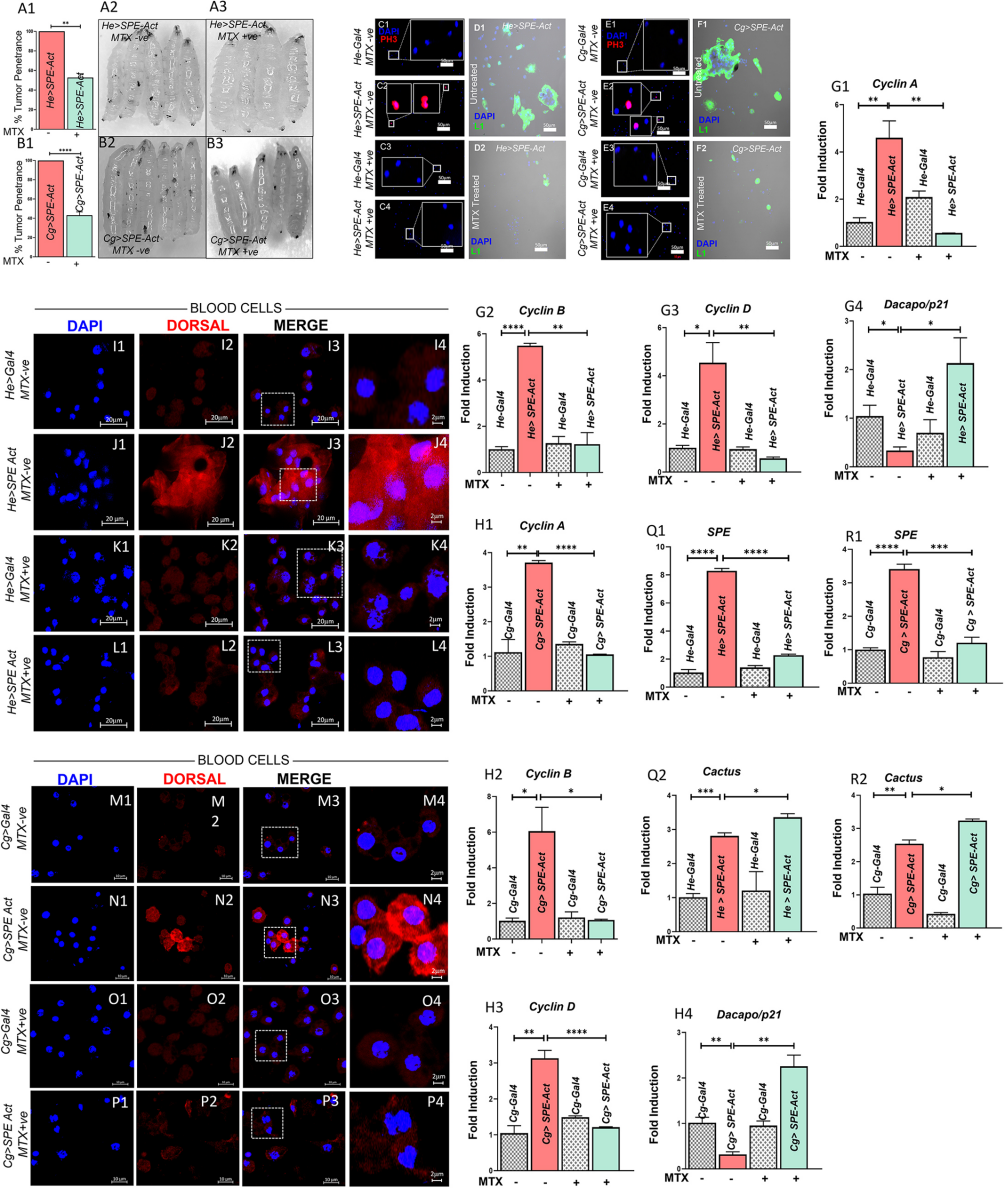
**Effect of MTX on the hematopoietic defects manifested by overexpression of *SPE* in larval immune tissues.** MTX treatment was studied for its impact on hematopoietic defects and pseudotumor formation in *He>SPE-Act* and *Cg>SPE-Act* larvae, characterized by overexpression of *SPE* in the immune tissues. (A1,B1) Percentage of pseudotumor penetrance in *He>SPE-Act* (A1) and *Cg>SPE-Act* (B1) larvae before and after MTX treatment. (A2,B2) Melanotic tumors were visible in the cuticle of untreated *He>SPE-Act* (A2) and *Cg>SPE-Act* (B2) larvae. (A3,B3) Significant reduction in pseudotumor penetrance and very small or no melanotic pseudotumors were observed after MTX treatment in the *He>SPE-Act* (A3) and *Cg>SPE-Act* (B3) larvae. Images were captured using a trinocular microscope with an attached Micaps ecocmos510B camera. (C1–F2) Anti-PH3 staining was used to identify mitotically active cells in the blood cells of third-instar wandering *He>SPE-Act* (C1–D2) and *Cg>SPE-Act* (E1–F2) larvae. Samples were stained with DAPI (nuclear stain, blue) and anti-PH3 (mitotically active cells, red) before and after MTX treatment. (C1,E1) Controls (*He-Gal4* and *Cg-Gal4*). (C2,E2) Untreated *He>SPE-Act* (C2) and *Cg>SPE-Act* (E2) larvae with increased mitotic activity. (C3,E3) MTX-treated control larvae. (C4,E4) MTX-treated *He>SPE-Act* (C4) and *Cg>SPE-Act* (E4) larvae with reduced mitotic activity. The effect of MTX treatment on lamellocyte lineage and cell cycle regulation was investigated in *He>SPE-Act* and *Cg>SPE-Act* larvae overexpressing *SPE*. (D1,F1) L1-positive blood cells (green, anti-L1 staining) in untreated *He>SPE-Act* (D1) and *Cg>SPE-Act* (F1) larvae, which are specific to the lamellocyte lineage, with nuclei counterstained by DAPI (blue). (D2,F2) Reduction in L1-positive blood cells was observed after MTX treatment in *He>SPE-Act* (D2) and *Cg>SPE-Act* (F2) larvae. Images were obtained using confocal microscopy (LSM710), with data from three biological replicates (*N*=3, *n*=12). (G1–G4) Transcript levels of *Cyclin A* (G1), *Cyclin B* (G2), *Cyclin D* (G3) and *dacapo* (*p21*; G4) in whole larvae (third instar), with and without MTX treatment and overexpression of *SPE* in the background of *He-Gal4*. (H1–H4) Transcript levels of *Cyclin A* (H1), *Cyclin B* (H2), *Cyclin D* (H3) and *dacapo* (*p21*; H4) in whole larvae (third instar), with and without MTX treatment and overexpression of *SPE* in the background of *Cg-Gal4*. Graphs show data from three biological replicates (*N*=3, *n*=50), analyzed using GraphPad Prism software version 8.0.2, with statistical significance determined by Student's *t*-test (unpaired, two-tailed); *P<0.05, **P<0.01, ****P<0.0001. (I1–P4) Dorsal nuclear localization was also assessed in the blood cells of third-instar wandering larvae from *He>SPE-Act* (I1–L4) and *Cg>SPE-Act* (M1–P4) backgrounds, with and without MTX treatment. Larvae were stained with DAPI (nuclear stain, blue), anti-Dorsal (Dorsal-specific antibody, red) and polymerized F-actin (cytoskeleton, green). (I1–L4) Images show untreated control *He-Gal4* (I1–I4), untreated *He>SPE-Act* (J1–J4), MTX-treated *He-Gal4* (K1–K4), and MTX-treated *He>SPE-Act* (L1–L4) larvae in the *He>SPE-Act* background. (M1–P4) Images show untreated *Cg-Gal4* (M1–M4), untreated *Cg>SPE-Act* (N1–N4), MTX-treated *Cg-Gal4* (O1–O4) and MTX-treated *Cg>SPE-Act* (P1–P4) larvae in the *Cg>SPE-Act* background. Data were obtained from three biological replicates (*N*=3, *n*=12), with confocal microscopy images captured under identical settings using the LSM710 system. (Q1–R2) Transcript levels of *SPE* (Q1,R1) and *Cactus* (Q2,R2) were quantified in whole third-instar larvae, with and without MTX treatment, in the context of overexpression of *SPE* in the *He-Gal4* (Q1,Q2) and *Cg-Gal4* (R1,R2) backgrounds. Data were obtained from three biological replicates (*N*=3, *n*=50), and all analyses were performed using GraphPad Prism version 8.0.2. Statistical significance determined by Student's *t*-test (unpaired, two-tailed); *P<0.05, **P<0.01, ***P<0.001, ****P<0.0001.

The Toll pathway can be activated by external infection or stress (Small et al., 2012; Lemaitre et al., 1996; De Gregorio et al., 2002; Jang et al., 2006). Here, to investigate the effects of MTX on immune cell dysfunction in *Drosophila* larvae, we overexpressed SPE, a positive regulator of the Toll pathway. We utilized two distinct GAL4 promoters, *Cg-Gal4* (expressed in both blood cells and fat body) and *Hemese-Gal4* (expressed only in blood cells), to drive SPE expression. We studied the hyperactive Toll pathway in larvae with overexpression of SPE via the UAS-GAL4 system, the classical approach to investigate tissue-specific overexpression or knockdown (Brand and Perrimon, 1993; Hasan et al., 2005). The efficacy of SPE overexpression was confirmed by measuring *SPE* transcript levels, which were significantly elevated in *SPE-Act* larvae (*He>SPE-Act*, 8.29-fold; *Cg>SPE-Act*, 3.41-fold) relative to those in untreated control larvae (Fig. 3Q1,R1). This SPE overexpression model also serves as a model of chronic inflammation, with 100% of larvae overexpressing SPE-Act exhibiting melanized masses (using the drivers *Cg-Gal4* and *He-Gal4*), which were not observed in wild-type larvae. MTX treatment significantly reduced pseudotumor formation to 43.21% in *He>SPE-Act* larvae and 52.81% in *Cg>SPE-Act* larvae compared to that in untreated controls (Fig. 3A1–A3, B1–B3). To further elucidate the impact of MTX on blood cell proliferation and differentiation, we assessed mitotic activity [via anti-phospho-histone H3 (anti-PH3)] and lamellocyte numbers (via anti-L1). In untreated *He>SPE-Act* and *Cg>SPE-Act* larvae, mitotic activity and lamellocyte concentrations were both significantly elevated (Fig. 3C2,E2). MTX treatment resulted in a significant reduction in both the mitotic cells and the lamellocyte numbers, consequently reducing blood pseudotumor counts (Fig. 3C4,E4,D2,F2). Because of the effects of MTX on mitosis, we next analyzed the transcript levels of cell cycle regulators and found that, in untreated *Cg>SPE-Act* whole larvae, *Cyclin A*, *Cyclin B* and *Cyclin D* were substantially upregulated, and that MTX treatment attenuated their expression (Fig. 3G1–G3,H1–H3), like we had observed during infection. Conversely, the expression of *dacapo* (the *Drosophila* homolog of *p21*), a cell cycle inhibitor, was downregulated in untreated *Cg>SPE-Act* larvae and upregulated following MTX treatment (Fig. 3G4,H4), suggesting that MTX impedes tumorigenesis by modulating cell division and cycle regulators. To further assess the influence of MTX on the Toll pathway, we analyzed the localization of Dorsal, the *Drosophila* homolog of NF-κB. In untreated *GAL4*-driven *SPE-Act* larvae (*He>SPE-Act* and *Cg>SPE-Act*), Dorsal displayed nuclear localization along with cytoplasmic expression, indicative of Toll pathway activation (Fig. 3J1–J4,N1–N4). However, MTX treatment inhibited nuclear localization of Dorsal, and we observed only cytoplasmic expression of Dorsal (Fig. 3L1–L4,P1–P4), which is consistent with Toll pathway suppression. These results are consistent with the idea that MTX suppresses hyperactive Toll signaling in *He>SPE-Act* and *Cg>SPE-Act* animals. In line with this interpretation, we also found a significant increase in Cactus levels in *He>SPE-Act* and *Cg>SPE-Act* in MTX-treated larvae (Fig. 3Q2,R2).

We verified the effects of MTX on Dorsal nuclear localization in *Cg>Toll^10b^* animals showing similar chronic inflammatory effects of hyperactive Toll signaling. In MTX-treated *Cg>Toll^10b^* animals, we observed simultaneous and significant rescue of tumor penetrance and reduction in Dorsal nuclear localization in blood cells of *Cg>Toll^10b^* animals compared to the untreated controls (Fig. S3A1–F). Collectively, these findings suggest that MTX treatment reduces chronic inflammation by (1) inhibiting cell cycle defects associated with overproliferation in our chronic inflammation model and (2) inhibiting Toll signaling.

### MTX rescues hematopoietic defects in Toll pathway hyperactivation *Ubc9^−/−^* mutants

**Fig. 4.**
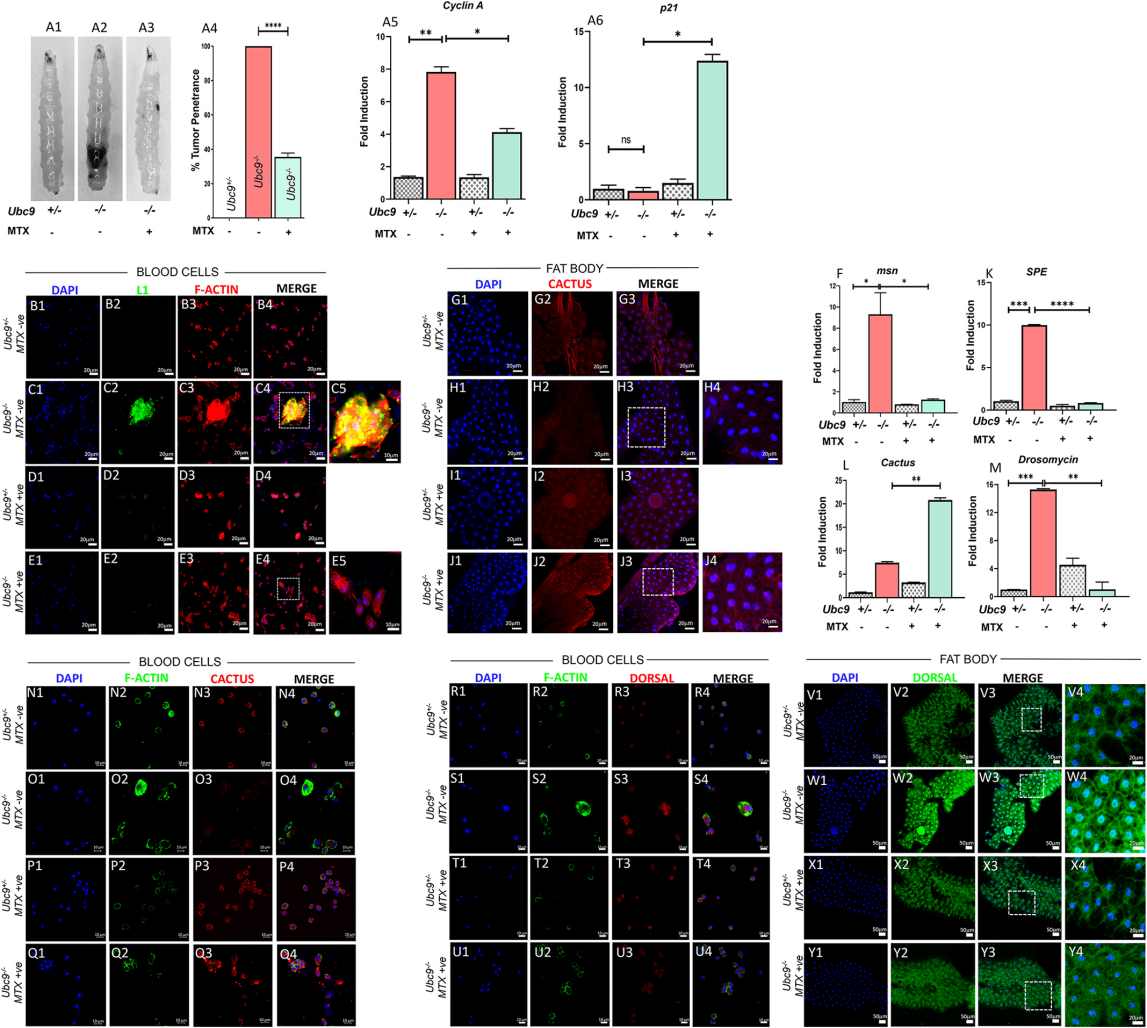
**Impact of MTX on dysregulated Toll/NF-κB pathway in *Ubc9^−/−^* third-instar larvae.** Ubc9 is an E2 conjugase and essential component of the SUMO pathway that regulates immune signaling, including the Toll/NF-κB pathway in fruit flies. This pathway controls the transcription factor Dorsal, which translocates to the nucleus to activate immune defense genes. In *Ubc9^−/−^* mutants, the absence of Ubc9 disrupts SUMOylation, impairing Dorsal nuclear translocation and deregulating immune responses. The effects of MTX treatment on the *Ubc9^−/+^* (heterozygote) and *Ubc9^−/−^* (mutant) third-instar larvae were evaluated in terms of pseudotumor penetrance and gene expression regulation. (A1–A3) Images of untreated *Ubc9^−/+^* (A1), untreated *Ubc9^−/−^* (A2) and MTX-treated *Ubc9^−/−^* larvae (A3) are shown. (A4) Pseudotumor penetrance between untreated *Ubc9^−/+^* control, untreated *Ubc9^−/−^* and MTX-treated *Ubc9^−/−^* larvae were quantified and represented as a percentage. (A5,A6) Gene expression levels of cell cycle regulators (*Cyclin A*; A5) and the cell cycle inhibitor dacapo (*p21*; A6) in *Ubc9^−/+^* control and *Ubc9^−/−^* whole larvae, before and after MTX treatment, in the context of Toll/NF-κB pathway activation. Statistical significance was assessed by Student's *t*-test (unpaired, two-tailed); *P<0.05, **P<0.01, ****P<0.0001, for comparisons between uninfected untreated, infected, and infected plus MTX-treated groups. Data were obtained from three biological replicates (*N*=3, *n*=50+), and graphs were processed using GraphPad Prism version 8.0.2. (B1–E5) The localization of L1-positive lamellocytes in circulating blood cells of third-instar *Ubc9^−/−^* larvae was marked through immunostaining. Larvae were stained with DAPI (nuclear stain, blue), anti-L1 (lamellocyte, green) and polymerized F-actin (cytoskeleton, red). Images show untreated *Ubc9^−/+^* heterozygote larvae (B1–B4), untreated *Ubc9^−/−^* larvae (C1–C5), MTX-treated *Ubc9^−/+^* heterozygote larvae (D1–D4) and MTX-treated *Ubc9^−/−^* larvae (E1–E5). Data were obtained from three biological replicates (*N*=3, *n*=12), and control and experimental images were captured under identical settings using confocal microscopy (LSM710). (F) Transcript levels of *msn* (lamellocyte marker) in *Ubc9^−/−^* whole larvae before and after MTX treatment. Statistical significance was determined by Student's *t*-test (unpaired, two-tailed); *P<0.05. Graphs were processed using GraphPad Prism version 8.0.2. (G1–J4) Cactus protein levels in the fat body cells of third-instar *Ubc9^−/−^* mutant larvae, with and without MTX treatment, was analyzed using confocal microscopy. Larvae were stained with DAPI (nuclear stain, blue) and anti-Cactus (Cactus-specific antibody, red). Images show untreated *Ubc9^−/+^* larvae (G1–G3), untreated *Ubc9^−/−^* larvae (H1–H4), MTX-treated *Ubc9^−/+^* larvae (I1–I3) and MTX-treated *Ubc9^−/−^* larvae (J1–J4). Data were collected from three biological replicates (*N*=3, *n*=12), and control and experimental images were captured under identical settings using confocal microscopy (LSM710). (K–M) Transcript levels of *SPE* (K), *Cactus* (L) and *Drosomycin* (M) in *Ubc9^−/−^* mutants before and after MTX treatment. Statistical significance was assessed by Student's *t*-test (unpaired, two-tailed); **P<0.01, ***P<0.001, ****P<0.0001. Data were processed using GraphPad Prism version 8.0.2. *N*=3, *n*=50. (N1–Q4) Cactus protein in the circulating blood cells of third-instar *Ubc9^−/+^* and *Ubc9^−/−^* larvae, with and without MTX treatment. Larvae were stained with DAPI (nuclear stain, blue), anti-Cactus (Cactus-specific antibody, red) and polymerized F-actin (cytoskeleton, green). Images show untreated *Ubc9^−/+^* larvae (N1–N4), untreated *Ubc9^−/−^* mutant larvae (O1–O4), MTX-treated *Ubc9^−/+^* larvae (P1–P4) and MTX-treated *Ubc9^−/−^* larvae (Q1–Q4). Data were obtained from three biological replicates (*N*=3, *n*=12), and immunostaining images were taken under identical settings using an LSM710 system. (R1–U4) As *Ubc9^−/−^* mutants have a disrupted *Ubc9* gene, which affects various immune-related signaling pathways, we assessed the impact of MTX treatment in these *Ubc9^−/−^* mutants. We stained larvae with DAPI (nuclear stain, blue), anti-Dorsal (specific to Dorsal protein, red in hemocytes and green in fat body cells), and polymerized F-actin (cytoskeleton, green in hemocytes). Circulating blood cells were imaged in untreated *Ubc9^−/+^* larvae (R1–R4), untreated *Ubc9^−/−^* larvae (S1–S4), MTX-treated *Ubc9^−/+^* larvae (T1–T4) and MTX-treated *Ubc9^−/−^* mutant larvae (U1–U4). (V1–Y4) Similarly, fat body cells were imaged in untreated *Ubc9^−/+^* larvae (V1–V4), untreated *Ubc9^−/−^* mutant larvae (W1–W4), MTX-treated *Ubc9^−/+^* larvae (X1–X4) and MTX-treated *Ubc9^−/−^* larvae (Y1–Y4). Data were obtained from three biological replicates (*N*=3), with 12 larvae per experimental group (*n*=12), and all confocal images were acquired using an LSM710 system under identical settings.

To confirm our findings, we investigated the effects of MTX on Toll pathway overactivation using a different model. We employed loss-of-function (LOF) mutants of *Ubc9* (also known as *lwr*), a negative regulator of the Toll pathway (Chiu et al., 2005; Huang et al., 2005; Kalamarz et al., 2012; Paddibhatla et al., 2010). MTX treatment significantly reduced the incidence of melanotic bodies in *Ubc9^−/−^* mutants, with penetrance decreasing from 100% to 35.55% (Fig. 4A1–A3,A4). Because *Ubc9^−/−^* mutants exhibit excessive blood cell proliferation, we determined whether the reduction in pseudotumor penetrance was attributable to MTX suppression of blood cell proliferation. To address this, we examined the expression of key cell cycle regulators [*Cyclin A* and *dacapo* (*p21*)] in third-instar whole larvae following MTX treatment. In untreated *Ubc9^−/−^* mutants, *Cyclin A* expression was dramatically elevated (7.83-fold), whereas in MTX-treated *Ubc9^−/−^* mutants, *Cyclin A* expression was elevated 4.12-fold, compared to that in untreated *Ubc9*^−/−^ mutants (Fig. 4A5). Conversely, *dacapo* expression, which inhibits cell cycle progression, was markedly upregulated in *Ubc9^−/−^* mutants treated with MTX (12.39-fold) and marginally upregulated in untreated controls (0.79-fold), compared to that in untreated *Ubc9^−/+^* heterozygotes (Fig. 4A6). To assess lamellocyte differentiation, we measured the presence of L1-positive cells in circulating hemolymph and evaluated *msn* gene expression, a marker of the lamellocyte lineage. Untreated *Ubc9^−/−^* mutants exhibited a significant increase in L1-positive lamellocytes (15.95%) compared to *Ubc9^−/+^* heterozygotes, which showed less than 1% lamellocyte populations (Fig. 4B1–B4,C1–C5). MTX treatment of the *Ubc9^−/−^* mutants reduced L1-positive cells to 2.27% (Fig. S2A), and *msn* expression was reduced from being 9.31-fold higher in untreated *Ubc9^−/−^* mutants than that in untreated *Ubc9*^−/+^ heterozygote siblings to being 1.27-fold higher than that in untreated *Ubc9*^−/+^ heterozygote siblings upon MTX treatment (Fig. 4F). There was no significant change in plasmatocyte populations, indicating that reduced lamellocyte population is not a consequence of general hematopoietic toxicity (Fig. S2B1–E4).

### MTX reduces chronic inflammation in *Ubc9^−/−^* mutants by downregulating NF-κB signaling

Next, we analyzed the impact of MTX on the expression of Toll/Dorsal pathway targets in *Ubc9^−/−^* LOF mutants. We first studied *SPE* and *Drosomycin* gene expression and found that levels of both genes were significantly elevated in the *Ubc9^−/−^* mutants (9.98-fold and 15.29-fold higher than those in untreated *Ubc9*^−/+^ heterozygotes, respectively), and that MTX treatment resulted in downregulation of both genes (to being 0.83-fold and 1.04-fold higher than those in untreated *Ubc9*^−/+^ heterozygotes, respectively) (Fig. 4K,M). Next, *Cactus* levels were assessed as the *Cactus* gene is also a transcriptional target of Toll/Dorsal signaling (Paddibhatla et al., 2010). We noticed that *Cactus* transcript levels were stable in *Ubc9^−/+^* heterozygotes but increased 20.8-fold, relative to those in untreated *Ubc9^−/+^* heterozygotes, in *Ubc9^−/−^* mutants following MTX treatment (Fig. 4L). Anti-Cactus antibodies confirmed increased Cactus expression in both the fat body and hemocytes via immunostaining in *Ubc9^−/−^* mutants after MTX treatment (Fig. 4H1–J4,O1–Q4). To examine whether MTX treatment can modulate the status of Toll/Dorsal signaling by inhibiting nuclear localization of the Dorsal transcription factor, we examined cytoplasmic and nuclear localization of Dorsal in *Ubc9^−/+^* heterozygotes and *Ubc9^−/−^* mutants in untreated and MTX-treated conditions. Immunostaining results revealed that, in untreated *Ubc9^−/−^* mutants, Dorsal was both nuclear and cytoplasmic in both immune tissues, whereas in *Ubc9^−/+^* heterozygotes, Dorsal remained cytoplasmic (Fig. 4R4,S4,V4,W4). MTX treatment inhibited Dorsal nuclear localization in *Ubc9^−/−^* mutants (Fig. 4S1–S4,U1–U4; Fig. S2F). In conclusion, taken together, these results indicate that MTX alleviates hematopoietic defects in *Ubc9^−/−^* mutants by modulating the expression of key Toll pathway components (*SPE*, *Cactus* and *Drosomycin*), affecting Cactus localization and inhibiting Dorsal nuclear localization. Thus, MTX demonstrates the potential to alleviate chronic inflammatory phenotypes in *Ubc9^−/−^* mutants by effectively inhibiting the Toll pathway.

### MTX ameliorates fat body abnormalities in *Ubc9^−/−^* mutants through inhibition of dysregulated insulin signaling

**Fig. 5.**
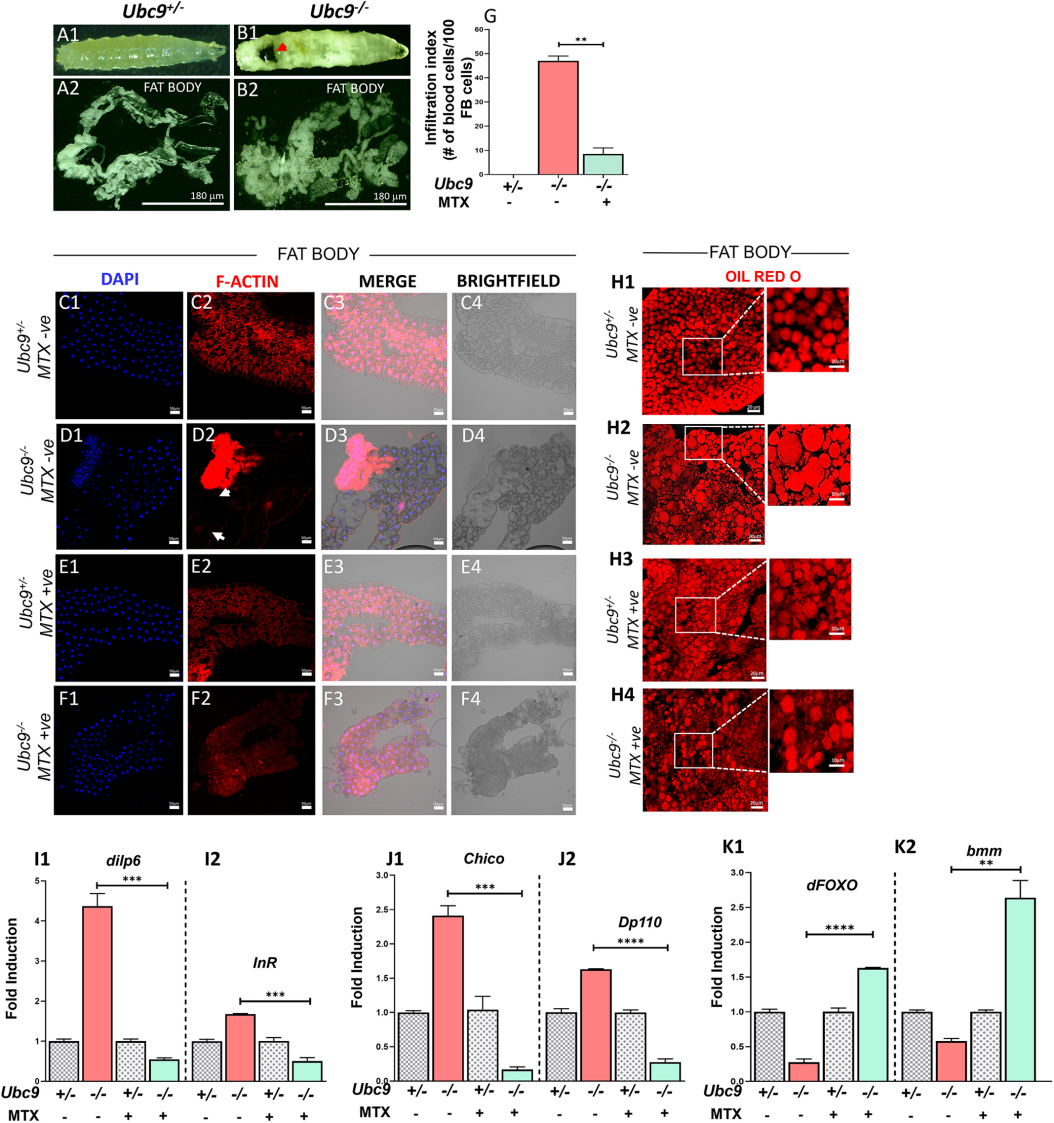
**Status of the nutritional pathway in *Ubc9^−/−^* animals.** (A1,A2) A third-instar *Ubc9^−/+^* larva (A1) and its total dissected fat content (A2). (B1,B2) A third instar *Ubc9^−/−^* mutant larva (B1) with a pseudotumor phenotype (red arrowhead) and its total dissected fat content (B2). (C1–F4) Fat body tissues dissected and stained with DAPI (nuclear stain, blue) and phalloidin (polymerized F-actin, red), along with brightfield images, in untreated *Ubc9^−/+^* larvae (C1–C4), untreated *Ubc9^−/−^* mutant larvae (D1–D4), MTX-treated *Ubc9^−/+^* larvae (E1–E4) and MTX-treated *Ubc9^−/−^* larvae (F1–F4). White arrows indicate infiltration of the fat body by blood cells. (G) Quantification of blood cells infiltrating fat body (FB) cells in untreated *Ubc9^−/−^* mutants compared to MTX-treated *Ubc9^−/−^* mutants, with *Ubc9^−/+^* heterozygotes serving as the control. Data were analyzed using GraphPad Prism version 8.0.2. Statistical significance was assessed by Student's *t*-test (unpaired, two-tailed); **P<0.01. (H1–H4) Untreated *Ubc9^−/+^* larvae (H1), untreated *Ubc9^−/−^* mutant larvae (H2), MTX-treated *Ubc9^−/+^* larvae (H3) and MTX-treated *Ubc9^−/−^* larvae (H4) stained with Oil Red O (specific to triglycerides, red) to assess stored triglyceride levels in the fat body. (I1–K2) Transcript levels of key components in the insulin signaling pathway, including the ligand *dilp6*, receptor *dInR*, receptor substrate *chico*, activated PI3K component *Dp110*, transcription factor *dFOXO* and ATGL homolog *brummer* (*bmm*) in third-instar *Ubc9^−/−^* whole larvae before and after MTX treatment. Data were analyzed using GraphPad Prism version 8.0.2. Statistical significance was assessed by Student's *t*-test (unpaired, two-tailed): **P<0.01, ***P<0.001, ****P<0.0001. *N*=3, *n*=50.

Given that the fat body cells are integral to pseudotumor formation in *Ubc9^−/−^* mutants (Paddibhatla et al., 2010) and are infiltrated by blood cells, we sought to investigate the potential effects of MTX on both the infiltrating hemocytes and adipose content. Consistent with previous observations, the fat body in *Ubc9^−/−^* LOF mutants displayed considerable disintegration (Paddibhatla et al., 2010), accompanied by increased fat content (Fig. 5B1,B2), compared to that in *Ubc9^−/+^* heterozygote siblings (Fig. 5A1,A2). In these mutants, the fat body underwent significant infiltration by blood cells, resulting in tissue degradation (Fig. 5D1–D4, white arrows indicate the infiltration). MTX treatment reduced the infiltration of blood cells into the fat body of *Ubc9^−/−^* mutants (Fig. 5F1–F4), without affecting adipose tissue in the *Ubc9^−/+^* heterozygotes (Fig. 5C1–C4,E1–E4). We quantified the infiltration index by counting the blood cells present in the fat body before and after MTX treatment (Fig. 5G). Furthermore, we assessed the size and number of fat globules in *Ubc9^−/−^* mutants stained with Oil Red O. These mutants exhibited significantly larger lipid globules (Fig. 5H2) than those of controls. However, in MTX-treated *Ubc9^−/−^* mutants, the size of lipid globules was comparable to that observed in *Ubc9^−/+^* heterozygotes (Fig. 5H1,H3,H4). Irregularities in polymerized F-actin staining indicating the abnormal cellular membrane, the disintegration of fat tissue and elevated triglyceride content prompted us to investigate further nutritional signaling in *Ubc9^−/−^* mutants. We questioned whether disturbances in the interplay between the immune pathway in the fat body and insulin signaling known to influence cellular growth, metabolism and tumorigenesis could take route via inflammatory responses. We, therefore, theorized a potential link between hyperactive Toll pathway-triggered inflammatory phenotypes (excessive blood cell proliferation, pseudotumorigenesis) to dysregulated insulin signaling in these *Ubc9^−/−^* mutants. Hence, we speculated that disruptions in the Toll pathway exacerbate the pathological effects on insulin signaling, particularly in the context of chronic inflammation and uncontrolled cell proliferation as observed in *Ubc9^−/−^* mutants. Previous studies in the contexts of obesity, type-2 diabetes and cancer have established intricate relationships between inflammatory pathways and nutritional regulation (Cohen and LeRoith, 2012; Scully et al., 2020), with *D. melanogaster* showing homologous pathways to those found in mammals. To examine the impact of *Ubc9* loss on insulin signaling, we measured the mRNA expression levels of key insulin signaling components – including the insulin ligand *dilp6* (also known as *Ilp6*), the receptor *dInR*, the substrate *chico*, the PI3K component *Dp110* (also known as *Pi3K92E*), the transcription factor *dFOXO* (also known as *foxo*) and the lipase *bmm* – in *Ubc9^−/−^* mutants with active Toll pathway signaling. We found that transcript levels of *dilp6* and *dInR* were significantly elevated, with a 4.3-fold and 1.68-fold increase, relative to those in untreated *Ubc9^−/+^* heterozygotes, respectively (Fig. 5I1,I2). Concurrently, the levels of *chico* and *Dp110* were upregulated 2.41-fold and 1.63-fold, relative to those in untreated *Ubc9^−/+^* heterozygotes, respectively (Fig. 5J1,J2), whereas *dFOXO* and *bmm* exhibited a marked decrease, with levels upregulated only 0.28-fold and 0.58-fold relative to those in untreated *Ubc9^−/+^* heterozygotes, respectively (Fig. 5K1,K2). These findings suggest that the increased triglyceride content observed in *Ubc9^−/−^* larval adipocytes could be attributed to enhanced insulin signaling, which downregulates *dFOXO* and *bmm*, both of which are involved in regulating lipid metabolism (Chakrabarti and Kandror, 2009; Zhang et al., 2012). Following MTX treatment, we assessed the expression of insulin signaling components and observed that the elevated levels of *dilp6*, *dInR*, *chico* and *Dp110* were significantly downregulated, while the lower levels of *dFOXO* and *bmm* were markedly upregulated (Fig. 5I1–K2). These results collectively imply that MTX normalizes triglyceride metabolism in *Ubc9^−/−^* mutants by suppressing the dysregulated nutritional signaling pathway.

### Genetic rescue of *Ubc9^−/−^* mutants

**Fig. 6.**
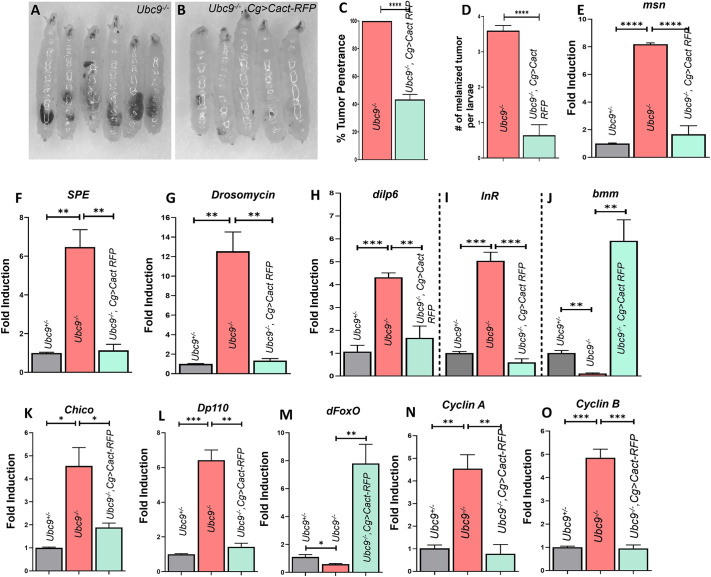
**Restoring the *Ubc9^−/−^* defects via genetic rescue by overexpressing Cactus.** (A) Third-instar *Ubc9^−/−^* mutant larvae with melanotic pseudotumors. (B) Third-instar *Ubc9^−/−^* larvae rescued genetically by overexpressing Cactus using the *Cg-Gal4* driver. (C,D) Percentage pseudotumor penetrance (C) and pseudotumor expressivity (D), comparing *Ubc9^−/−^* mutants to *Ubc9^−/−^* mutants with genetic rescue. Statistical significance was determined by Student's *t*-test (unpaired, two-tailed); ***P<0.0001. Graphs were processed using GraphPad Prism version 8.0.2. *N*=3, *n*=50. (E–G) Transcript levels of *msn* (E), *SPE* (F) and *Drosomycin* (G) in third-instar whole *Ubc9^−/−^* larvae and *Ubc9^−/−^* larvae with genetic rescue, with *Ubc9^−/+^* heterozygote larvae serving as the control. Statistical significance was calculated using Student's *t*-test (unpaired, two-tailed); **P<0.01, ****P<0.0001. Graphs were processed using GraphPad Prism version 8.0.2. *N*=3, *n*=50. Experiments were conducted at 25°C. (H–M) Transcript levels of insulin signaling components – *dilp6* (H), *dInR* (I), *bmm* (J), *chico* (K), *Dp110* (L) and *dFOXO* (M) – in third-instar *Ubc9^−/−^* larvae and *Ubc9^−/−^* larvae with genetic rescue. *Ubc9^−/+^* larvae served as the control. Statistical significance was assessed by Student's *t*-test (unpaired, two-tailed); *P<0.05, **P<0.01, ***P<0.001. Graphs were processed using GraphPad Prism version 8.0.2. *N*=3, *n*=50. Experiments were conducted at 25°C. (N,O) Gene expression of cell cycle regulators *Cyclin A* (N) and *Cyclin B* (O) in third-instar *Ubc9^−/−^* larvae and *Ubc9^−/−^* larvae with genetic rescue, with *Ubc9^−/+^* larvae as the control. Statistical significance was determined by Student's *t*-test (unpaired, two-tailed); **P<0.0, ***P<0.001. *N*=3, *n*=50+. Graphs were processed using GraphPad Prism version 8.0.2.

We know from earlier literature (Paddibhatla et al., 2010) that Cactus levels in *Ubc9^−/−^* mutant fat body are significantly reduced. To examine whether overexpression of Cactus rescues *Ubc9* defects, we generated *Ubc9^−/−^* mutants overexpressing wild-type Cactus (*UAS-Cactus-RFP* in *Ubc9^−/−^* background), using the *Cg-Gal4* driver. This model provided the opportunity to explore whether the mechanisms underlying Cactus overexpression rescue of *Ubc9* defects are similar to those conferred by MTX treatment and whether hyperactivation of the Toll signaling pathway influences insulin signaling, and to elucidate a possible mechanistic link, We compared the phenotypes of mutants (*Ubc9^−/−^*) and rescued (*Ubc9^4-3/5^, Cg>Cact-RFP*) larvae (Fig. 6A,B). We assessed pseudotumor penetrance (the percentage of larvae exhibiting melanotic masses) and expressivity (the number of pseudotumors per larva) in both conditions. Genetic rescue resulted in a significant reduction of penetrance by 56.55% (Fig. 6C) and a decrease in tumor expressivity from 3.59 pseudotumors per mutant larva to 2.95 pseudotumors per rescued larva (Fig. 6D). Supporting these observations were the results obtained from qRT-PCR experiments, with *msn* (a marker of lamellocyte lineage) transcript levels elevated 8.19-fold in *Ubc9^−/−^* mutants and only 1.66-fold in the rescued larvae, relative to those in untreated *Ubc9^−/+^* heterozygotes, supporting the decrease in blood tumors primarily composed of lamellocytes (Fig. 6E). Furthermore, expression of the Toll pathway components and downstream targets, *SPE* and *Drosomycin*, was upregulated in *Ubc9^−/−^* mutants (6.48-fold and 12.55-fold, relative to that in untreated *Ubc9^−/+^* heterozygotes, respectively), with only 1.14-fold and 1.35-fold increases in expression relative to that in untreated *Ubc9^−/+^* heterozygotes, respectively, in the rescued animals (Fig. 6F,G). Given the rectified Toll pathway in the rescued larvae, we further examined the transcript expression of insulin signaling components. As anticipated, *Ubc9^−/−^* mutants exhibited upregulated insulin signaling, with *dilp6*, *dInR*, *chico* and *Dp110* levels elevated 4.32-fold, 5.04-fold, 4.55-fold and 6.41-fold, relative to those in *Ubc9^−/+^* heterozygotes, respectively, while levels of *bmm* and *dFOXO* were significantly reduced to being only 0.11-fold and 0.58-fold higher than those in *Ubc9^−/+^* heterozygotes, respectively. The transcript levels were similar in the rescued larvae (*Ubc9^−/−^, Cg>Cact-RFP*) and *Ubc9*^−/+^ heterozygotes, corroborating that Toll pathway inhibition via Cactus overexpression normalizes insulin signaling (Fig. 6H–M), thereby stabilizing triglyceride levels in *Drosophila* adipocytes. Moreover, we observed dysregulated cell cycle dynamics in the *Ubc9^−/−^* mutants, with upregulated *Cyclin A* and *Cyclin B*. In the genetic rescue background, these Cyclin levels were normalized to those of *Ubc9*^−/+^ heterozygotes, indicating restoration of blood cell proliferation regulation via proper cell cycle control (Fig. 6N,O).

In conclusion, the results obtained from the MTX and genetic rescue experiments in *Ubc9^−/−^* mutants are comparable in indicating effective suppression of the Toll pathway. Additionally, our study highlights that abnormal insulin signaling in these mutants contributes to the observed fat body defects associated with loss of Ubc9. Collectively, these results support the hypothesis that MTX treatment can restore immune homeostasis in *Ubc9^−/−^* mutants by concurrently modulating both Toll pathway activation and dysregulated insulin signaling.

## DISCUSSION

**Fig. 7.**
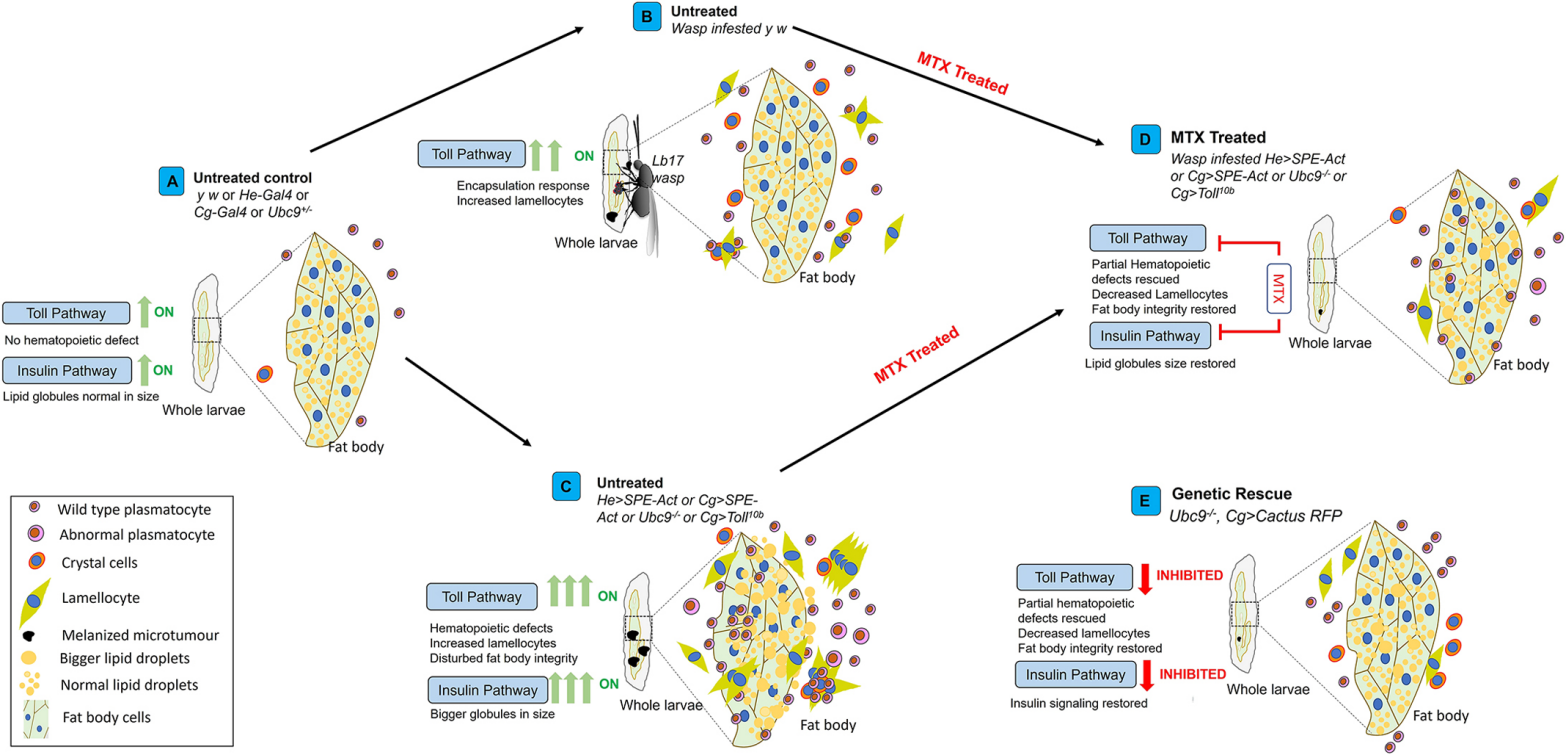
**Summary schematic.** (A) Schematic representation of the fat body and blood cell distribution in wild-type *Drosophila* third-instar larvae (*y w*, *He-Gal4*, *Cg-Gal4* or *Ubc9^−/+^*), which do not exhibit hematopoietic defects. Both Toll and insulin signaling pathways are at basal levels in these control larvae. (B) *Lb-17* female wasp invading the wild-type *y w* third-instar *Drosophila* larval host by injecting its egg into the hemocoel. This invasion triggers the host's encapsulation response via activation of the Toll pathway. (C) A schematic of fat body and blood cells in *SPE* overexpression models (*He>SPE-Act* and *Cg>SPE-Act*), *Ubc9^−/−^* and *Cg>Toll^10b^*, showing constitutive activation of the Toll pathway. This results in hematopoietic defects and high fat body infiltration by blood cells, accompanied by larger fat globules due to enhanced insulin signaling. (D) The effects of MTX treatment in these models, in which hyperactive Toll pathway signaling in wasp-infested, *He>SPE-Act* and *Cg>SPE-Act*, *Ubc9^−/−^* and *Cg>Toll^10b^* mutants is mitigated, restoring fat body integrity and reducing fat globule size due to decreased insulin signaling. (E) A schematic of the genetic rescue of *Ubc9^−/−^* mutants by introducing an extra copy of Cactus. This genetic rescue results in partial but significant improvements, including reduced pseudotumor formation and lowered Toll pathway activation.

In our study, we used *Drosophila* as a tool to investigate the potential effects of MTX on immune metabolism under the control of constitutively activated Toll pathway. Our findings support the role of MTX in restoration of immune homeostasis by downregulating the overactive Toll signaling in *Ubc9^−/−^* mutants. The immune defects observed in larvae due to overstimulated Toll pathway (aberrant blood cell development, compromised fat body integrity, pseudotumor formation and immune disruption) are rectified by MTX treatment. Our research is the first to show the suppressive effects of MTX on hyperactivation of the Toll/NF-κB pathway. This is evident by reduced nuclear localization of Dorsal in hemocytes and fat cells, changed expression of Toll pathway target genes (*Drosomycin*, *SPE*, *Cactus*), decreased fat body infiltration and enhanced regulation of insulin signaling (Figs 1–5 and 7) post MTX treatment. Whereas MTX suppressed *SPE* and *Drosomycin*, we observed increased expression of *Cactus* in *Ubc9^−/−^* mutants post MTX treatment (Figs 2–4), which was unexpected given that *Cactus* is also a target of Dorsal (Paddibhatla et al., 2010). It is possible that *Cactus* transcript levels are increased as a compensatory mechanism and a response to regulate the Toll signaling pathway via maintaining the complex with Dorsal in cytoplasm upon MTX treatment. Also, the transcriptional regulation of *Cactus* independent of Dorsal transcription factor can provide valuable insights into Toll pathway regulation. Furthermore, published literature from mammalian studies revealed that MTX is involved in stabilizing IκBα (mammalian homolog of *Drosophila* Cactus) protein by suppressing its phosphorylation (Majumdar and Aggarwal, 2001). Thus, the stabilization of Cactus protein we observed (Fig. 2H2,P2, Fig. 3J2,Q2,R2) could be a direct effect of MTX treatment, further supporting the increased stabilization of Dorsal in cytoplasm and lower nuclear localization. Elucidating how MTX affects either the transcriptional regulation of *Cactus* or its protein stability in *Drosophila* can help us to understand its mechanism of action. We also observed an increase in *Cyclin B* expression post MTX treatment in uninfected larvae (Fig. 2), suggestive of unknown effects of MTX. The effect of MTX on cyclin (*Cyclin B*) without any wasp infection in control larvae (*y w*) could be a response to the larval growth and the developmental stage. Further studies focused on the effects of MTX on cell cycle regulators (in larval background with overexpression of cell cycle regulators resulting in hematopoietic defects) would provide valuable insights.

The genetic rescue of *Ubc9^−/−^* defects with overexpression of Cactus in *Ubc9^−/−^* mutants restored immune balance, illustrating comparable results to MTX effects on the activated Toll pathway (Figs 6 and 7). Similar observations have been made with other anti-inflammatory agents, such as aspirin, which alleviated inflammatory phenotypes in the Toll pathway and *hop^Tum-l^* mutants (Panettieri et al., 2019). Although earlier findings such as MTX anti-cancer (Yadav et al., 2021) and aspirin anti-inflammatory (Panettieri et al., 2019) effects, along with our current findings, provide evidence of significant improvements in the immune tissue (blood cells and fat body), a complete phenotypic rescue was not achieved in any scenario. Hence, further research into combination therapies that can help investigate focused immune tissue defects could prove advantageous. Although newly established pharmacological agents are regularly tested in research and clinical trials, reassessing their efficacy on hallmarks of cancer and their potential side effects is equally crucial. The consequences of extensive examination of the novel anti-cancer molecules often yields inconclusive data or side effects that cannot be ignored, emphasizing the significance of repurposing conventional drugs to alleviate side effects while improving therapeutic effectiveness. In conclusion, our research underscores (1) the repurposing of MTX, an anti-cancer drug, to address immune defects associated with Toll pathway activation; (2) the impact of the Toll and insulin pathways on developmental, hematopoietic and immune defects; and (3) the restoration of immune homeostasis in *Ubc9^−/−^* mutants through genetic rescue via Cactus overexpression, which mitigates hyperactive Toll and insulin signaling. Previous studies have established that hyperactive Toll pathway, both systemically and in the fat body, suppresses insulin signaling, supporting immune defense over growth and reproduction in *r4>Toll^10b^* (*r4-Gal4* is expressed in the fat body) larvae and adults (DiAngelo et al., 2009). In contrast, our results show that hyperactivation of the Toll/NF-κB pathway in both the blood cells and the fat body of *Cg>Toll^10b^* animals (*Cg-Gal4* is expressed in both the immune tissues) disturbs fat body metabolism and elevates insulin signaling by upregulating the pathway components specifically in third-instar larvae with high metabolic demands preceding pupariation. Taken together, these results suggest that the effects of hyperactive Toll on insulin signaling are tissue and stage specific, varying between the immune tissues and developmental contexts. Prospective studies can focus on the direct mechanistic link between the Toll pathway and insulin signaling, providing an opportunity for therapeutic intervention.

Future investigations should also explore the potential interplay between folate and MTX in larvae rescued post MTX treatment, providing insights into the mechanisms underlying the effects of MTX. Because MTX exerts its effects through inhibition of dihydrofolate reductase (DHFR) and subsequent folate metabolism, an avenue for further investigation is whether folate supplementation could reverse these effects. Given that MTX and folate share a competitive relationship at the metabolic level, it is reasonable that folate could counteract the therapeutic benefits of MTX by restoring key folate-dependent pathways, including those involved in nucleotide synthesis, cell division and immune regulation. Such competition for the same metabolic targets, such as the tetrahydrofolate pool, can reduce the efficacy of MTX treatment and potentially reverse its immunosuppressive and anti-inflammatory actions. However, if MTX is not exercising its effects via the conventional anti-folate mechanism and instead influences immune pathways through non-folate-dependent mechanisms (e.g. by modulating Toll signaling or disrupting metabolic homeostasis), the addition of folate could have a minimal or distinct impact on the MTX-induced changes. In such a scenario, understanding the specific metabolic and signaling pathways activated by MTX, independent of folate antagonism, would be critical. Investigating these alternative pathways could reveal novel insights into the wider pharmacological potential of MTX, especially in the milieu of autoimmune or inflammatory disorders.

### Implications

Drug repositioning and repurposing expediate the discovery of novel therapeutic applications for existing medications, providing a more convenient and cost-effective path to treatment development (Kulkarni et al., 2023). By leveraging the established safety profiles of these drugs, researchers can significantly reduce the time and resources typically required for new drug discovery (Pushpakom et al., 2019). This approach is especially important while addressing the unattended medical requirements, particularly in multifaceted pseudotumors that are very challenging to treat with the existing therapies owing to resistance shown by the growing cancer cells (Ashburn and Thor, 2004). MTX, an anti-cancer drug, is investigated for its immunomodulatory properties, and this opens new avenues for researchers, specifically pharmacologists who are addressing inflammatory diseases via drug repositioning and repurposing.

Our study provides an experimental model for evaluating the effects of repurposed anti-cancer drugs. Also, the signaling pathways contributing to deterioration of immune tissues, such as insulin signaling, play a critical role in metabolic dysfunctions and tumorigenesis, making them valuable targets for therapeutic intervention in diseases such as diabetes and cancer (Saltiel and Kahn, 2001). By regulating insulin signaling, drugs such as MTX also alleviate metabolic disturbances, improving immune homeostasis and thereby enhancing the effectiveness of established cancer treatments. This approach provides significant potential for the investigation of combined therapeutic strategies that synergistically associate metabolic reprogramming with immunotherapeutic interventions, thus facilitating targeted cancer treatments.

## MATERIALS AND METHODS

A standard protocol of cornmeal–malt–agar mixed media was implemented for breeding of all fly stocks, which were maintained at 25°C under 12 h: 12 h light–dark cycles.

### Fly stocks

#### Wild type

*Drosophila* wild-type *y w* (Professor Shubha Govind's laboratory, City College, City University of New York, New York, NY).

#### Mutants

*hop^Tum-l^*, gain-of-function mutant of the JAK/STAT pathway [a gift from Professor Shubha Govind's laboratory (Panettieri et al., 2019; Piper et al., 2014)]; *Ubc9* [*Drosophila* Ubc9 is encoded by a gene also known as *lesswright* (*lwr*) or *semushi* (Chiu et al., 2005)]; *Ubc9^5^ FRT40/CyO y+* and *y w*; *Ubc9^4-3^/CyO y+* (a gift from Professor Shubha Govind's laboratory) (Chiu et al., 2005). *Ubc9^4-3^*/*Ubc9^5^* trans heterozygote mutants showed extended larval development whereby most of them died by day 10 as larvae (Paddibhatla et al., 2010).

#### *Ubc9^4-3^/CyO Tb*, *Ubc9^5^ FRT40/CyO Tb* and *UAS-SPE-Activated/CyO Tb* stock preparation

For ease of selection between homozygous lethal mutants (*Ubc9^4-3^*/*Ubc9^5^ FRT40*) and heterozygote siblings (*Ubc9^5^ FRT40*/*CyO y+*, *Ubc9^4-3^*/*CyO y+*) at larval stages, *Ubc9^4-3^*/*CyO Tb* and *Ubc9^5^ FRT40*/*CyO Tb* strain was prepared by cross-breeding with the stocks carrying *CyO Tb* marker (*CyO Tb* marker stocks were a gift from Dr Rakesh Kumar Mishra's laboratory, Centre for Cellular and Molecular Biology, Hyderabad, India). *CyO Tb* marker-carrying larvae are short and stout at larval stages compared to non-*CyO Tb*-carrying larvae. The homozygous mutant stock (*Ubc9^4-3^*/*Ubc9^5^ FRT40*) showed both melanized and non-melanized pseudotumors. Melanized pseudotumors were visible under the cuticle, whereas non-melanized pseudotumors were transparent. *Ubc9^4-3^*/*CyO Tb* and *Ubc9^5^ FRT40*/*CyO Tb* heterozygotes did not show any defects and were used as control larvae.

#### UAS lines

*UAS-SPE-Activated* (*UAS-SPE-Act*) [amino acids 135–400, from B. Lemaitre (Jang et al., 2006)]; *w; Ubc9^5^, Cg>Cact-RFP, UAS-Toll^10b^* [*Toll^10b^*, gain-of-function mutant of the Toll pathway (a gift from Professor Shubha Govind's laboratory)]. *Toll^10b^* carries a missense point mutation in the cytoplasmic domain of the *Drosophila* Toll receptor, specifically altering a single amino acid residue critical for conformational regulation (Anderson et al., 1985).

#### GAL4 lines

*Collagen-Gal4* (*Cg-Gal4*; Cg is ubiquitously expressed in the fat body, circulating hemocytes and some cells of the lymph gland) (Asha et al., 2003; a gift from Professor Shubha Govind's laboratory); *Hemese-Gal4* [*He-Gal4*; Hemese is expressed exclusively by blood cells and hematopoietic tissue (Kurucz et al., 2003)].

### Parasitoid wasps

A standard protocol (Sorrentino et al., 2002) was followed for rearing *Leptopilina boulardi*-*17* (*Lb-17*; strain 17) wasps (Schlenke et al., 2007) on the wild-type fly *y w* strain. Infection was performed on 3-day-old larvae (after 72 h of egg laying).

### MTX treatment in wasp infestation experiment

*Drosophila* wild-type (*y w*) flies were kept in a chamber for 12 h at room temperature for egg laying. After 48 h, the first-instar larvae were transferred to a 60 mm Petri dish containing fly food mixed with MTX (1 ml of drug was added to 3 ml of fly food). This was referred to as the first MTX treatment. After 12 h, late second-instar larvae were exposed to their parasitoid wasps, *Lb-17*, for 12 h, and then wasps were removed. After 36 h, early third-instar larvae were transferred to a similar Petri dish containing fly food mixed with MTX (1 ml of drug was added to 3 ml of drug). This was referred to as the second MTX treatment. Then, after 24 h, all the third-instar host larvae were collected and killed. All the larvae of the control group that were uninfected, with or without MTX treatment, successfully pupariated and eclosed without any developmental defects. Encapsulation index was calculated using protocol as described in Yadav et al. (2021).

### MTX treatment in hematopoietic mutants

*Ubc9^4-3^*/*Ubc9^5^* or *Ubc9^−/−^* mutant flies were kept in a chamber for 12 h for egg laying. After 48 h, first-instar larvae were transferred to a 60 mm Petri dish containing fly food mixed with MTX (1 ml of drug was added to 3 ml of fly food) (first MTX treatment). After 60 h, the early third-instar larvae were transferred to another 60 mm Petri dish containing fly food mixed with MTX (1 ml of drug was added to 3 ml of fly food) (second MTX treatment). Then, after 24 h, all the third-instar heterozygote (*Ubc9^−/+^*) and mutant (*Ubc9^−/−^*) larvae were collected and killed. Heterozygotes (*Ubc9^−/+^*) were considered as controls and used for comparison with (*Ubc9^−/−^*) mutants. All the larvae of the control group (*Ubc9^−/+^*) with or without MTX treatment successfully pupariated and eclosed without any developmental defects.

### MTX treatment of larvae with overexpression of *UAS-SPE-Act*

Two crosses were set up for the overexpression experiments with two separate drivers, *He-Gal4* and *Cg-Gal4*, i.e. *w; He-Gal4* crossed to *w; UAS-SPE-Act/CyO Tb* and *w; Cg-Gal4* crossed to *w; UAS-SPE-Act/CyO Tb*. Both these crosses were kept in a chamber for 12 h for egg laying. After 48 h, larvae were transferred to a 60 mm Petri dish containing fly food mixed with MTX (1 ml of drug was added to 3 ml of fly food) (first MTX treatment). After 60 h, early third-instar larvae were transferred to another 60 mm Petri dish containing fly food mixed with MTX (1 ml of drug was added to 3 ml of fly food) (second MTX treatment). Then, after 24 h, all the third-instar experimental larvae (*Non-Tb, He>SPE-Act* and *Non-Tb, Cg>SPE-Act*) were collected and killed. *He-Gal4* and *Cg-Gal4* larvae were used as experimental controls in this experiment. All the larvae of the control group with or without MTX treatment successfully pupariated and eclosed without any developmental defects.

#### Genetic rescue experiments

To perform the genetic rescue in *Ubc9^−/−^* mutants, we incorporated and overexpressed a wild-type copy of Cactus (negative regulator of Toll pathway) tagged with RFP via *Cg-Gal4*. The genetic cross was set up between *Ubc9^5^, Cg-Gal4>Cact-RFP*/*CyO y*^+^ and *Ubc9^4-3^*/*CyO Tb* (*CyO Tb* marker stocks were a gift from Dr Rakesh Kumar Mishra's laboratory). *CyO Tb* marker-carrying larvae are short and stout at larval stages (and curly wings at adult stage) compared to *Non*-*CyO Tb* larvae. All the F1 generation larvae with the *CyO Tb* phenotype were eliminated, and all the *Non-CyO Tb* flies were selected for the genetic rescue stocks (*Ubc9^5^, Cg-Gal4>Cact-RFP*/*Ubc9^4-3^*). It was carrying the overexpressed Cactus via *Cg-Gal4* that resulted in significant reduction in the size of melanized pseudotumors compared to that in *Ubc9^−/−^* mutants. Thus, the stock was used for further experimental analysis.

### Penetrance expressivity, encapsulation and infiltration index

After the second MTX treatment in *Ubc9^−/−^* LOF mutants, the third-instar larvae were observed under a microscope to document the pseudotumors visible through the cuticle for determining the penetrance, and these larvae were further dissected to score the total number of melanized tumors, along with the non-melanized pseudotumors, to determine the expressivity per larva. Fat bodies of the control and mutant larvae were dissected. The dissected fat body was fixed, blocked and stained with Alexa Fluor^®^ 555 phalloidin along with the nuclear counterstain DAPI, then mounted in 50% glycerol. The numbers of single blood cells adhered/fat body cell were documented with the help of an axial fluorescence microscope. Aggregates of blood cells on the fat body were omitted from documentation. Data were analyzed by GraphPad Prism 8.0.2 tool, and Student’s *t*-test (unpaired, two-tailed) was utilized for statistical analysis. A similar procedure was followed as mentioned above to determine pseudotumor penetrance in overexpression of *UAS-SPE-Act* using *Collagen-Gal4* and *Hemese-Gal4* drivers.

### Immunostaining, microscopy, data collection and analysis

12-15 third-instar developmentally synchronized larvae were dissected for fat bodies, hemolymph and lymph gland after cleaning with 1× PBS, double distilled water, 70% ethanol and again with 1× PBS to avoid contamination. Samples were fixed with 4% paraformaldehyde (PFA) at room temperature for 15 min. Fixed samples were given quick washes with 1× PBS followed by 30 min of incubation with 3% bovine serum albumin (BSA) for blocking. Samples were washed again with 1× PBS and incubated overnight (at 4°C) with primary antibodies in 1× PBS+3% BSA+0.2% Triton X-100. Primary antibodies were as follows: mouse monoclonal anti-dorsal [74A, 1:10; Developmental Studies Hybridoma Bank (DSHB), Biogenuix Medsystem Pvt Ltd (Whalen and Steward, 1993)]; mouse monoclonal anti-Cactus [3H12, 1:10, DHSB, Biogenuix Medsystem Pvt Ltd (Whalen and Steward, 1993)]; lamellocyte-specific mouse monoclonal anti-L1/Atilla [1:100, gift from Dr Istvan Ando (Kurucz et al., 2007)]; plasmatocyte-specific mouse monoclonal anti-Nimrod C [1:100, gift from Dr Istvan Ando, (Kurucz et al., 2007)]; goat polyclonal anti-Relish (sc-26912, 1:50, Santa Cruz Biotechnology) and rabbit polyclonal anti-phospho-histone H3 (ser10) [9701, 1: 600, Cell Signaling Technology (CST)]. Washed samples were incubated for 3 h 45 min at room temperature with commercially available secondary antibody conjugated with Alexa Fluor^®^ 488-anti mouse IgG (4408, 1:200, CST) and/or Alexa Fluor^®^ 488-anti rabbit IgG (4412, 1:200, CST) and/or Alexa Fluor^®^ 555-anti-mouse IgG (4409, 1:200, CST) and/or Alexa Fluor^®^ 555-anti-rabbit IgG (4413, 1:200, CST). Samples were washed twice with 1× PBS and once with 1× PBS+ 0.2% Triton X-100 (1× PBST). Samples were incubated overnight (at 4°C) with Alexa Flour^®^ 488 Phalloidin (8878, 1:20, CST) and Alexa Flour^®^ 555 Phalloidin (8953, 1:20, CST) for polymerized F-actin staining. The following day, after three washes, the samples were counterstained for 15 min at room temperature with the nuclear dye DAPI (4083, 2 µg/ml, CST) or Hoechst 33342 (4082, 2.5 µg/ml, CST) as the final step for staining procedure. Final slides were mounted in 50% glycerol. Imaging was performed using a Carl Zeiss Laser scanning confocal microscope (LSM710) at 20× and 40× (with immersion oil objectives). Raw images (CZI files) were taken and processed into final images (TIF files) using ZEN 3.1(Zen lite) blue edition software. Identical settings were used for obtaining control and experimental images.

### Dorsal count

Approximately eight to ten wandering third-instar larvae were dissected to remove the fat body and stained with anti-Dorsal antibody with counter nuclear stain DAPI. Three fields of view were taken into consideration for counting the total number of fat body cells (DAPI-positive cells) and the number of Dorsal nuclear-localized cells (anti-Dorsal colocalizing with DAPI). More than 280 cells from each (eight to ten larvae) were analyzed for each experimental setup. Three independent experiments were performed. Student’s *t*-test (unpaired, two-tailed) was performed for statistical analysis using GraphPad Prism 8.0.2.

### Pixel quantification

Pixel quantification was utilized for quantifying levels of Cactus protein. Using anti-Cactus antibody, fat body tissue was stained for Cactus protein. The intensity of the red fluorescent signal (pixels) was quantified using ZEN 3.1 (Zen lite) blue edition software. More than 300 fat body cells from each of 12 larvae were analyzed for each experimental setup. Three independent experiments were performed. Student’s *t*-test (unpaired, two-tailed) was performed for statistical analysis using GraphPad Prism 8.0.2. Controls were normalized, and only two experimental setups were compared.

### Oil Red O staining for triglycerides

Oil Red O staining was performed to stain triglycerides in the dissected fat body. Eight to ten larvae were thoroughly washed with 1× PBS. The entire fat body was gently taken out. Following treatments as indicated, the fat body tissues were washed twice with 1× PBS and fixed in 3.7% formaldehyde for 1 h at room temperature. After aspiration of the formaldehyde, the cells were stained with Oil Red O (23576, SRL Pvt Ltd.) for 1 h. Oil Red O was prepared by dissolving 0.5 g Oil Red O in 100 ml 2-propanol and diluting it with water (6:4), followed by filtration. Stained tissue was washed gently in PBS. Slides were imaged using the 20× and 40× lenses of a Carl Zeiss Laser scanning confocal microscope (LSM710).

### Sample preparation, RNA isolation and real time-PCR

More than 50 third-instar developmentally synchronized larvae were washed for RNA isolation (TRIzol method, Invitrogen). RNA concentration was quantified by a Nanodrop 2000 spectrophotometer (Thermo Fisher Scientific). Approximately 2.5 µg RNA was taken as template for kit-based cDNA synthesis (iscript TM, Bio-Rad Laboratories). Real-time quantitative PCR was performed running the standard step-one plus PCR program: 1.05 μl of the cDNA sample was mixed with KAPA SYBRR FAST Universal (006255-8-1, KAPA Biosystems) and primers to set up a 105 μl reaction mix. Transcript levels detected were normalized to *Rp49* (also known as *RpL32*) mRNA values.

Primer sequences used for real-time PCR were as follows: *Rp49* forward primer (5′-GACGCTTCAAGGGACAGTATCTG-3′), reverse primer (5′-AAACGCGGTTCTGCATGAG-3′); *Cyclin A* forward primer (5′-TCAGCGTGGGCACTGAAACGG-3′), reverse primer (5′-GGGCGATGTTTCTTCTCGCTCTCC-3′); *Cyclin B* forward primer (5′-GCCGAGGACGAGCACCATACG-3′), reverse primer (5′-GTGAGGCAGCTGCAATCTCCGA-3′); *Cyclin D* forward primer (5′-CAGCTTGCCTCTTACTGGCT-3′), reverse primer (5′-ACACTGCTCCCTTGCCATAC-3′); *dacapo* (*p21*) forward primer (5′-CCCGAGTCCTGAATCCTGTG-3′), reverse primer (5′-TGGAGCTACCGAAGAGGTCA-3′); *spätzle* forward primer (5′-GGAGCGGATCAACCCTGTG-3′), reverse primer (5′-TTGGATTATAGCTCTGCGGAAAG-3′); *SPE* forward primer (5′-CTTTTCGCTGATCGCATTTT-3′), reverse primer (5′-CACCGGATTTGTCCAGTTCT-3′); *Cactus* forward primer (5′-CTGCTCAACATCCAGAACGA-3′), reverse primer (5′-GCCGAACTTCTCTGTCAAGG-3′); *Drosomycin* forward primer (5′-ATCCTGAAGTGCTGGTGCGAAGGA-3′), reverse primer (5′-ACGTTCATGCTAATTGCTCATGG-3′); *msn* forward primer (5′-AAGGTGGGTCTCCGCAAATC-3′), reverse primer (5′-ATCAACCGCATGGAAACCCT-3′); *dilp6* forward primer (5′-TGCTAGTCCTGGCCACCTTGTTCG-3′), reverse primer (5′-GGAAATACATCGCCAAGGGCCACC-3′); *InR* forward primer (5′-GACGATGGCTACCCGATG-3′), reverse primer (5′-GCCTCCAATCAGGAAGATC-3′); *chico* forward primer (5′-GCAAGTTGTCATTCAA-3′), reverse primer (5′-ATCCCAAGACACTTTG-3′); *Dp110* forward primer (5′-GTCCACCTCCACAAGTCGAT-3′), reverse primer (5′-TGTGCAGCGTCAACTGAAAG-3′); *dFOXO* forward primer (5′-GGATGCGGAGTCGATGTCTT-3′), reverse primer (5′-CCCTTTATCCCAAATATGATGCCT-3′); *bmm* forward primer (5′-AATGGCGTCGAATCAGACTT-3′), reverse primer (5′-AACACAGATGGGGATTTGGA-3′).

### Statistics

All the documented data from the control and experimental samples were analyzed for statistical significance. To compare the characteristics between two groups of cohorts, we utilized Student's *t*-test (unpaired, two-tailed). Graphs with error bars show mean±s.e.m. *P*<0.05 was considered significant. Biological repeats (*N*), sample size (*n*) and Student's *t*-test results are mentioned in the figure legends. All the graphical data were analyzed using GraphPad Prism software (version 8.0.2).

## Supplementary Material

10.1242/joces.263816_sup1 Supplementary information
