# Supplementary material for: Methotrexate alleviates chronic inflammation in a Drosophila model
Source: J Cell Sci. 2025 Nov 3;138(21):jcs263816. doi: 10.1242/jcs.263816 (PMC12633795; doi:10.1242/jcs.263816)
Supplement: Supplementary information [file joces-138-263816-s1.pdf]

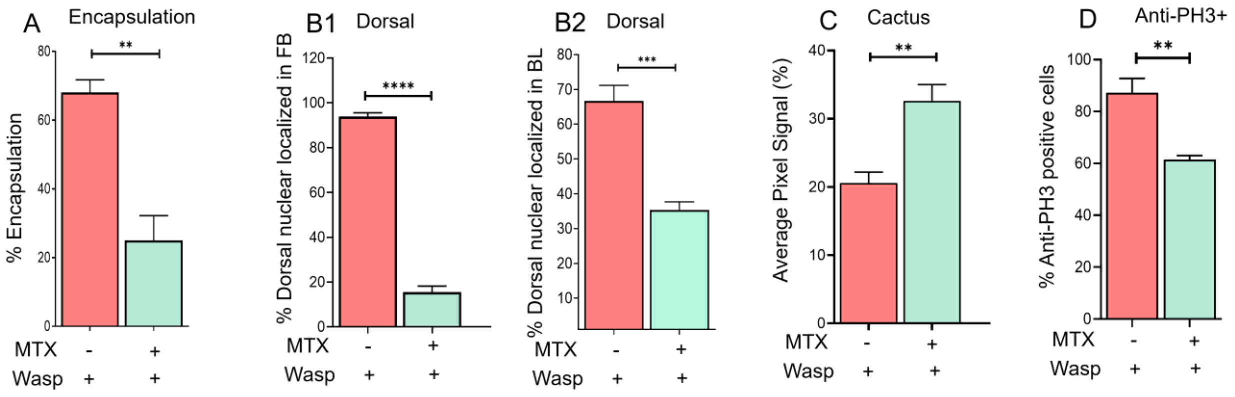

Fig. S1. (A) Graphical representation of the percentage of encapsulation in third instar larvae comparing untreated wasp infestation to MTX-treated wasp infestation. Percentage of Dorsal nuclear localization in untreated vs. MTX-treated wasp infestation in fat body, FB (B1) and blood cells, BL (B2). (C) Quantification of average pixel intensity of Cactus antibody staining in the fat body tissues between untreated and MTX-treated wasp-infested larvae. (D) Percentage of anti-PH3-positive cells in untreated vs. MTX-treated wasp infestation. Statistical significance was determined using Student's *t*-test (unpaired, two-tailed) with **$P < 0.0$, ***$P < 0.001$. N=3, n=50+. Graphs were processed using GraphPad Prism version 8.0.2.

Journal of Cell Science • Supplementary information

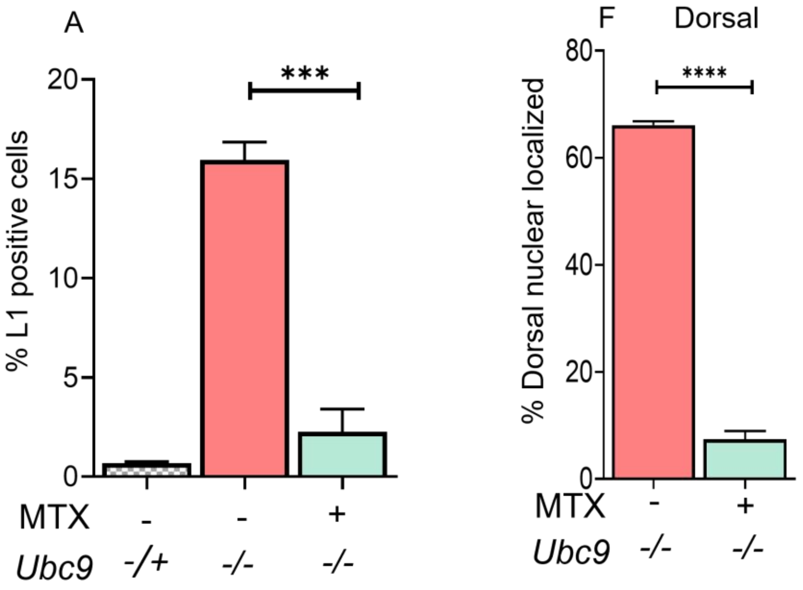
A
20
15
10
5
0
% L1 positive cells
***
MTX - - +
Ubc9 -/+ -/- -/-
F Dorsal
80
60
40
20
0
% Dorsal nuclear localized
****
MTX - +
Ubc9 -/- -/-

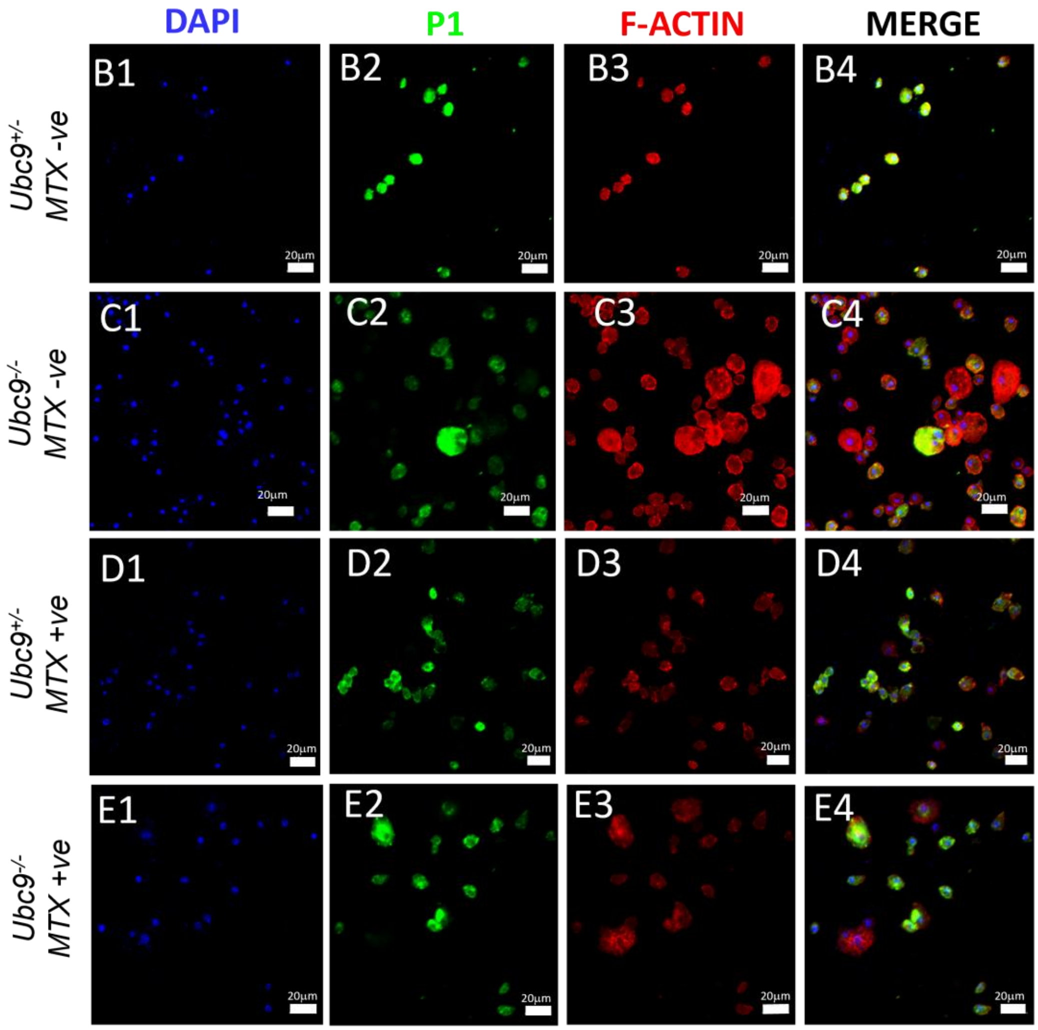
DAPI
P1
F-ACTIN
MERGE
B1
B2
B3
B4
Ubc9+/- MTX -ve
C1
C2
C3
C4
Ubc9-/- MTX -ve
D1
D2
D3
D4
Ubc9+/- MTX +ve
E1
E2
E3
E4
Ubc9-/- MTX +ve
20µm

Fig. S2. (A) Statistical analysis of the percentage of L1-positive cells in untreated vs. MTX-treated *Ubc9*$^{-/-}$ mutants, with *Ubc9*$^{-/+}$ heterozygotes serving as controls. (B–E) Representative confocal images of circulating blood cells from third instar *Ubc9*$^{-/-}$ stained for P1 (Plasmatocytes, green), polymerized F-actin (cytoskeleton, red), and DAPI (nuclear stain, blue):(B1–B4) untreated *Ubc9*$^{-/+}$, (C1–C4) untreated *Ubc9*$^{-/-}$, (D1–D4) MTX-treated *Ubc9*$^{-/+}$, and (E1–E4) MTX-treated *Ubc9*$^{-/-}$. N=3, n=12 (B–E). Control and experimental images were captured using identical settings in confocalmicroscopy (LSM710). (F) Statistical analysis of the percentage of dorsal nuclear-localized cells in untreated *Ubc9*$^{-/-}$ vs. MTX-treated *Ubc9*$^{-/-}$. Statistical significance was determined using Student's t-test (unpaired, two-tailed) with $^{**}P < 0.0$, $^{***}P< 0.001$. N=3, n=50+. Graphs were generated using GraphPad software version 8.0.2.

Journal of Cell Science • Supplementary information

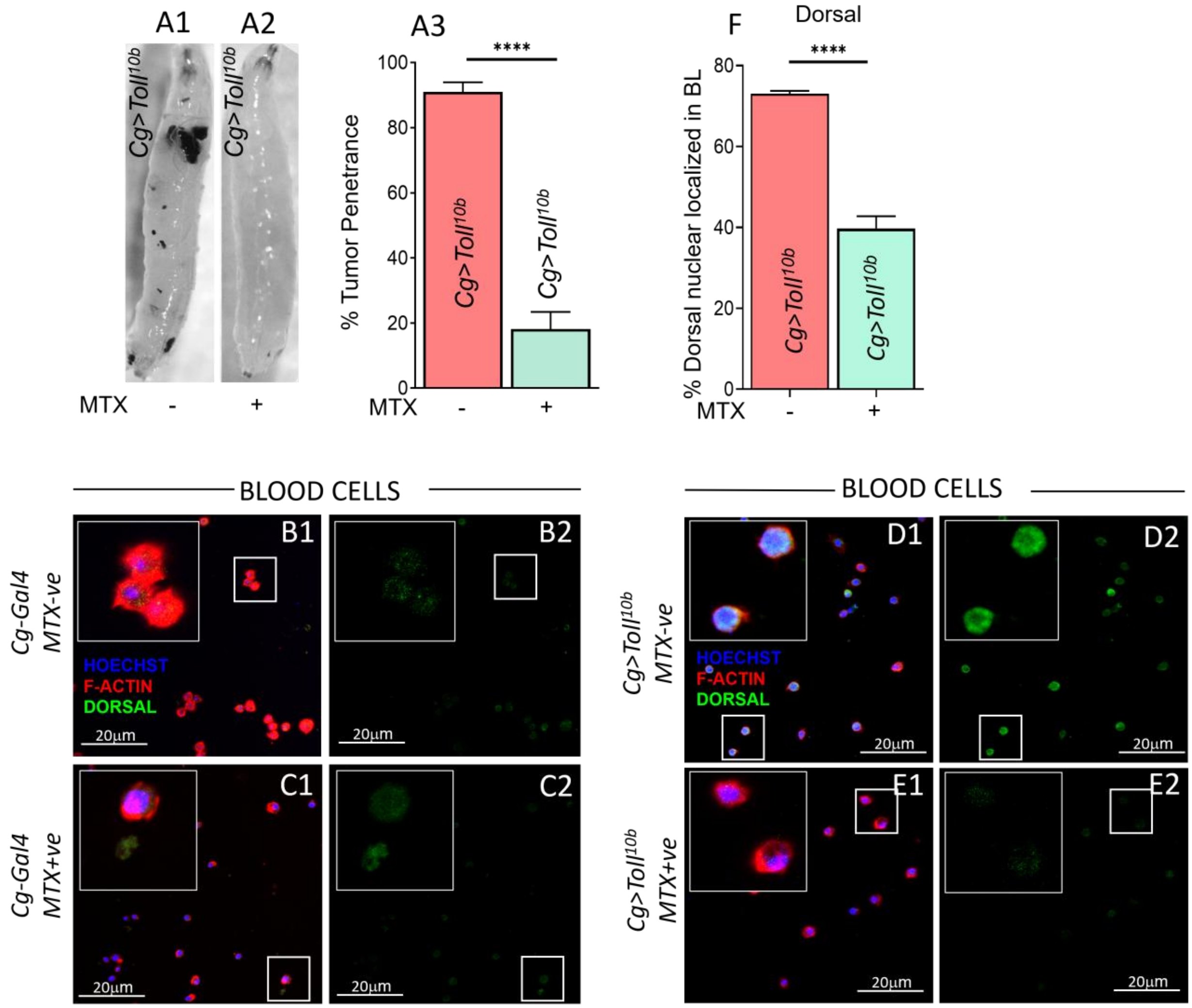

Fig. S3. MTX treatment was assessed for its impact on hematopoietic abnormalities and pseudotumor formation in *Cg>Toll^{10b}* larvae, characterized by active gain of function of *Toll^{10b}* in the immune tissues (*Toll^{10b}* allele is a well-characterized, constitutively active gain-of-function mutant of Toll pathway receptor in fruit flies (Anderson et al., 1985). Tumor penetrance in untreated and MTX-treated *Cg>Toll^{10b}* larvae was evaluated and compared. Representative images of untreated (A1) and MTX-treated (A2) *Cg>Toll^{10b}* larvae are shown, with quantification of pseudotumor penetrance expressed as a percentage (A3). Panels B1–E2 show confocal images of circulating hemocytes stained for Dorsal (green), polymerized F-actin (cytoskeleton, red), and Hoechst (nuclei, blue): untreated *Cg>Gal4* (B1–B2), MTX-treated *Cg>Gal4* (C1–C2), untreated *Cg>Toll^{10b}* (D1–D2), and MTX-treated *Cg>Toll^{10b}* (E1–E2). All confocal images were acquired using identical settings on a Leica SP8 microscope. Quantification of nuclear Dorsal localization in hemocytes is presented in panel F. Statistical analysis was performed using an unpaired, two-tailed *Student's t*-test; ***$P < 0.0001$. Sample sizes: N = 3 biological replicates, n ≥ 12–20 cells per group. Graphs were generated using GraphPad Prism (v8.0.2).
